# The Accumulative Effect of Multiple Postnatal Risk Factors with the Risk of Being Overweight/Obese in Late Childhood

**DOI:** 10.3390/nu16101536

**Published:** 2024-05-20

**Authors:** Ting Wu, Zijun Liao, Jing Wang, Mengjiao Liu

**Affiliations:** 1 School of Public Health, Jiangxi Medical College, Nanchang University, Nanchang 330006, China; wuting@email.ncu.edu.cn; 2 Jiangxi Provincial Key Laboratory of Disease Prevention and Public Health, Nanchang University, Nanchang 330006, China; 3 Capital Institute of Pediatrics, Beijing 100020, China; liaozijun43@163.com; 4 Murdoch Children’s Research Institute, The Royal Children’s Hospital, Parkville, VIC 3052, Australia; jing.wang@mcri.edu.au; 5 Department of Pediatrics, The University of Melbourne, Parkville, VIC 3010, Australia

**Keywords:** children, cohort, overweight/obesity, postnatal risk factors

## Abstract

Most past studies focused on the associations of prenatal risk factors with the risks of childhood overweight/obesity. Instead, more postnatal risk factors are modifiable, with less knowledge of their cumulative effects on childhood obesity. We analyzed data of 1869 children in an Australian birth cohort. Key postnatal risk factors included: maternal and paternal overweight/obesity during the child’s infancy, tobacco exposure, low family socioeconomic score, breastfeeding duration < 6 months, early introduction of solid foods, and rapid weight gain during infancy. The risk score was the sum of the number of risk factors. The primary outcome is overweight/obesity in late childhood (11–12 years); secondary outcomes are high-fat mass index (FMI), body fat percentage (BF%), and waist-to-height ratio (WHtR). Poisson regression models were used in the analyses. Children with higher risk scores had higher risks of overweight/obesity (*p*-for-trends < 0.001). After adjusting covariates, compared with those with 0–1 risk factors, children with 4–6 risk factors had 4.30 (95% confidence interval: 2.98, 6.21) times higher risk of being overweight/obesity; the relative risks for high FMI, BF%, and WHtR were 7.31 (3.97, 13.45), 4.41 (3.00, 6.50), and 6.52 (3.33, 12.74), respectively. Our findings highlighted that multiple postnatal risk factors were associated with increased risks of being overweight/obesity in late childhood.

## 1. Introduction

Childhood obesity is a significant public health concern around the world. Over the past two decades, the prevalence of obesity in children and adolescents has grown at an alarming pace worldwide, increasing from 0.7% to 5.6% for boys and 0.9% to 7.8% for girls, with around 50 million girls and 74 million boys with obesity [1]. Obesity since childhood predisposes individuals to various metabolic and cardiovascular comorbidities, including type 2 diabetes and hypertension [2]. Identifying modifiable risk factors early in life is, therefore, imperative for effective prevention.

The first 1000 days of life—from conception to the age of 24 months—are crucial for children’s development and prevention of childhood obesity [3]. Many prenatal and intrapartum factors were reported to increase the risk of obesity in children, such as maternal pre-pregnancy obesity [4], excessive gestational weight gain [5], maternal hyperglycemia [6], maternal smoking during pregnancy [7], and cesarean delivery [8]. Postnatal risk factors, identified in parents and children from birth to age two, are also crucial to the risk of childhood obesity and offer more opportunities for intervention. For example, breastfeeding for more than six months in comparison to less than six months was associated with a lower odds ratio (OR) of the “high body mass index (BMI) z-score” trajectory from ages 1 to 5 years (OR: 0.65, 95%CI: 0.43, 0.98) [9] and rapid weight gain during the first 1.5 years is a risk factor of subsequent obesity (OR: 2.94, 95%CI: 1.17, 7.43) [10].

Nevertheless, most previous studies have focused on analyzing risk factors individually within the first 1000 days rather than examining the potential collective effects on the likelihood of childhood overweight or obesity [11,12]. These factors often co-exist and could influence each other. For example, one study reported that a combination of four modifiable risk factors, including smoking during pregnancy, excessive gestational weight gain, breastfeeding less than 12 months, and less than 12 months of sleep per hour during infancy, predicted an obesity prevalence of 28%, compared with 4% in those without risk factors at ages 7 to 10 years [13]. It is necessary to assess if there is a need to assess the combined impact of postnatal risk factors on the risk of overweight/obesity, which aligns with the public health reality and will be more effective in terms of prevention [13]. 

This study leverages data from the Longitudinal Study of Australian Children (LSAC) and the LSAC’s Child Health CheckPoint (CheckPoint) wave to investigate the combined effects of postnatal risk factors associated with being overweight/obese in late childhood. Our findings aim to inform prevention efforts and guide public health policy and practice.

## 2. Materials and Methods

### 2.1. Study Design and Participants

Detailed information about LSAC’s study design and participant recruitment is mentioned elsewhere [14]. A population-representative sample of children aged 0–1 year was recruited into LSAC’s birth cohort with biennial data collection since 2004. Children who were born in 2004 were included and were at least six months old at the interview. Exclusions criteria were: (1) fewer than 20 active children within a postcode area, (2) non-permanent residents, or with a death date recorded according to the national Medicare system, and (3) individuals receiving alternative health services [15]. The response rate of the first invitation in 2004 was 57.2% (n = 5107), of which 73.7% (n = 3764) were reserved at wave 6 in 2014. 

The Birth cohort families who participated in the LSAC wave 6 home interview were eligible for the ChildHealth CheckPoint (CheckPoint) module. The CheckPoint was a detailed cross-sectional biophysical marker wave nested between LSAC waves 6 and 7, conducted between February 2015 and March 2016. Of the total LSAC wave 6 participants, 49.8% (n = 1874) of children aged 11–12 years participated in the CheckPoint. These children, along with their primary caregivers, underwent face-to-face interviews to facilitate data collection. Additional insights into the CheckPoint study design are available elsewhere for reference [16,17].

**Figure 1:**
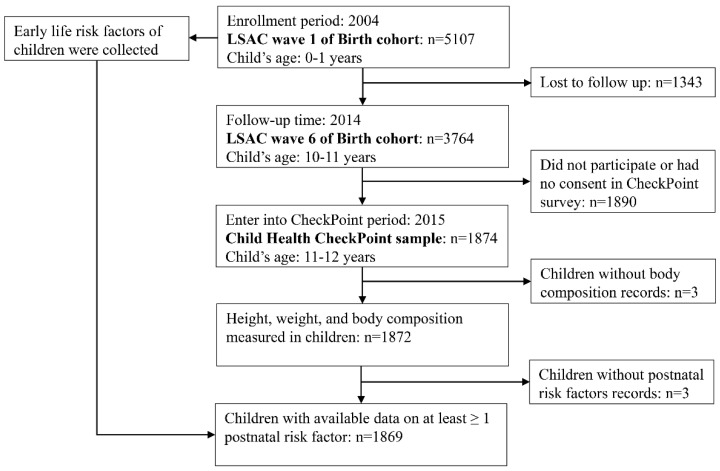
Flow chart of participants. LSAC: the Longitudinal Study of Australian Children. CheckPoint: Child Health CheckPoint.

For the current analysis, we included 1869 children who had available data on at least one postnatal risk factor as well as on at least one obesity index. Figure 1 describes the flow chart of study participants.

The CheckPoint study was approved by the Royal Children’s Hospital Melbourne Human Research Ethics Committee (33225D) and the Australian Institute of Family Studies Ethics Committee (14–26). A parent or guardian provided written consent for their own and their child’s participation in the study. 

### 2.2. Measures

#### 2.2.1. Exposures from LSAC

The LSAC team collected information through face-to-face structured interviews with children’s caregivers (95% were the child’s biological mother) [15]. Table S1 details questions related to the postnatal risk factor and other early life factors collected in LSAC wave 1. The key variables for this study were derived as follows:

Maternal and paternal overweight/obesity during the child’s infancy: mothers and fathers self-reported their weight and height during their child’s infancy, with parental BMI calculated as weight (kg) divided by height (m^2^). We defined maternal and paternal overweight/obesity during the child’s infancy as BMI ≥ 25 kg/m^2^, according to the World Health Organization (WHO) [18]. 

Breastfeeding < 6 months: the children’s breastfeeding duration was assessed by asking the mother “How old was the child (age in days) when he/she completely stopped being breastfed? (include expressed breastfeeding and not necessarily exclusively)”. Breastfeeding duration as <6 months (no distinction between exclusive and non-exclusive breast milk) was categorized based on the recommendation of the American Academy of Pediatrics [19]. 

Rapid weight gain (RWG) during infancy: Infant weight at wave 1 was measured on a Salter Australia scale by weighing the infant with an adult, then the adult alone, and subtracting the latter from the former. Age- and gender-specific weight-for-age z scores were calculated using the 2000 Centers for Disease Control and Prevention Growth Charts [20]. RWG during infancy was defined as an increase in the weight-for-age z score of ≥0.67, a threshold that has been previously reported to correlate with the increased risk for childhood obesity [21,22,23]. 

Tobacco exposure: the children’s primary caregiver was asked “Including yourself, how many people who live with you smoke inside the house?”. Tobacco exposure was considered positive if there were any smokers in the home during wave 1. 

Low family socioeconomic score (SES): the SES variable was derived from standardized scores for combined annual household income; parents’ years of education; and parents’ occupations [24]. The SES were categorized into normal (≥25th percentile) and low (<25th percentile) based on z-scores. 

Early introduction of solid foods: the introduction of solid foods was documented by asking the child’s primary caregiver “How old was the child when he/she first had solid food regularly? (Regularly = more than twice a week for several continuous weeks. Solid food = baby cereals, pureed fruits, etc.—not milk or drinks)”. The early introduction was defined as starting solids before the age of 4 months [25,26].

#### 2.2.2. Outcomes from LSAC’s CheckPoint Wave

Physical measurements were taken with children in light clothing, without shoes or socks. Height was recorded to the nearest 0.1 cm, and weight to the nearest 0.1 kg [27]. BMI was calculated as weight (kg) divided by height (m^2^). Body fat mass was measured by four-limb Bioelectrical impedance analysis (InBody230, Biospace, Seoul, Republic of Korea) by four-limb segmental body composition scales at assessment centers, or by two-limb body composition scales (Tanita BC-351, Kewdale, Australia) at home visits [28]. Body fat percentage (BF%) was calculated as body fat mass weight (kg) divided by body mass weight (kg) × 100. The fat mass index (FMI) was calculated as fat mass weight (kg) divided by height squared (m^2^). Waist circumference (cm) was measured horizontally around the navel by lifting the shirt or jumper and lowering the belt or waistband in children. Waist-to-height ratio (WHtR) was calculated as waist circumference (cm) divided by height (cm). 

Overweight/obesity in children was defined as having a BMI z-score ≥ 85th percentile according to the United States Centers for Disease Control and Prevention (CDC) [20]. Secondary outcomes included an FMI or BF% at or above the 75th percentile for age and sex as indicative of being overweight/obese [29,30], and abdominal obesity was defined by WHtR ≥ 0.5 [31,32].

#### 2.2.3. Covariates

The age of children and mothers was calculated to the nearest week from the date of birth (DOB) and the interview date of LSAC wave 1. The child’s DOB and sex were imported into LSAC from Medicare Australia’s enrolment database. Attending maternal DOB was self-reported by parent questionnaire. The mother’s age at conception was obtained by subtracting the child’s age from the mother’s age.

Additional covariates included birth weight (grams), gestational age (weeks), delivery type (caesarean section/vaginal birth), assisted reproductive technology for conception (yes/no), self-reported gestational diabetes (yes/no), and maternal smoking during pregnancy (yes/no) (Table S1).

### 2.3. Statistical Analysis

The outcome variable was a binary variable, which was defined as the presence or absence of overweight/obesity. The Poisson regression models with robust variance errors [33] were used to verify the independent association of postnatal risk factors (maternal and paternal overweight/obesity during the child’s infancy, tobacco exposure, low family SES, breastfeeding duration < 6 months, early introduction of solid foods, and RWG during infancy) with the likelihood of childhood overweight/obesity. Subsequently, risk factor scores were computed by summing each individual’s number of significant risk factors, identified in the initial analysis. 

The Poisson regression model with robust variance errors was applied to determine the relative risk of overweight/obesity among children with cumulative risk scores, using children with risk scores of 0–1 as the reference group. Model 1 was adjusted for maternal age at conception, child’s sex and age at measurement, and birth weight. To examine whether these associations could be explained by prenatal factors, model 2 was further adjusted for the child’s gestational age, delivery type (caesarean section/vaginal birth), assisted reproductive technology for conception, maternal smoking during pregnancy, maternal gestational diabetes, and child’s age of solid foods introduction. To quantify the predicted probability of overweight/obesity, we employed parameter estimates derived from multivariate logistic regression, fixing non-modifiable factors at mean values of the cohort’s maternal age and child’s birth weight to represent a “typical” participant profile.

The additive- and multiplicative-scale interaction measures were assessed to examine the interaction effects between pairs of risk factors on childhood overweight/obesity. The attributable proportion (AP), relative excess risk due to interaction (RERI), and synergy index (SI) were computed to elucidate additive interactions, thereby determining whether combined exposures increased risk beyond the sum of single effects. Multiplicative interactions were evaluated to ascertain if the combined risk factors augmented the risk beyond their product [34].

We conducted sensitivity analyses using multiple imputation by chained equations to account for missing data, creating 20 imputed datasets. These datasets were integrated and analyzed in Stata using MI ESTIMATE to ensure the robustness of our findings. 

All statistical analyses were performed in R 4.3. The statistical significance level is defined as *p* < 0.05.

## 3. Results

Sample characteristics are shown in Table 1. Of the included children, the means of maternal age, maternal BMI, and paternal BMI were 31.4 [standard deviation (SD) 4.8] years, 25.2 (SD 5.1) kg/m^2^, and 26.8 (SD 3.8) kg/m^2^, respectively; nearly half of the children were girls (49.0%), and the mean age of children was 11.5 (SD 0.5) years; 36.0% had breastfeeding duration < 6 months, and 7.7% had solid foods introduction < 4 months. The mean BMI, FMI, BF%, and WHtR of children were 19.2 (SD 3.4) kg/m^2^, 4.4 (SD 2.6) kg/m^2^, 21.7 (SD 8.4) %, and 0.4 (SD 0.1), respectively.

In the analyses of the single risk factor, maternal overweight/obesity [RR: 2.37 (95% confidence interval (CI): 1.97, 2.87)], paternal overweight/obesity [RR: 1.77 (1.39, 2.25)], tobacco exposure [RR: 1.76 (1.36, 2.29)], breastfeeding duration < 6 months [RR: 1.24 (1.03, 1.48)], RWG [RR: 1.54 (1.26, 1.89)], and low family SES [RR: 1.51 (1.27, 1.79)] was individually and significantly associated with the risk of overweight/obesity in children aged 11–12 years. These risk factors are similarly related to high FMI, BF%, and WHtR, except that the breastfeeding duration < 6 months was not associated with BF%, and early introduction of solid foods was not statistically associated with any of the four children’s overweight/obesity outcomes (Table S2). Therefore, we calculated the postnatal risk factor scores based on six factors except for the early introduction of solid foods. A total of 942 children had available data on all the risk factors and were included in the subsequent analyses: 354 (37.6%) children had zero or one postnatal risk score, 273 (29.0%) had two risk scores, 187 (19.8%) had three risk scores, and 128 (13.6%) had four or more risk scores. Characteristics of study participants according to the postnatal risk factor scores are shown in Table S3. Most characteristics were comparable between the included and the non-included children (Table S4).

**Figure 2:**
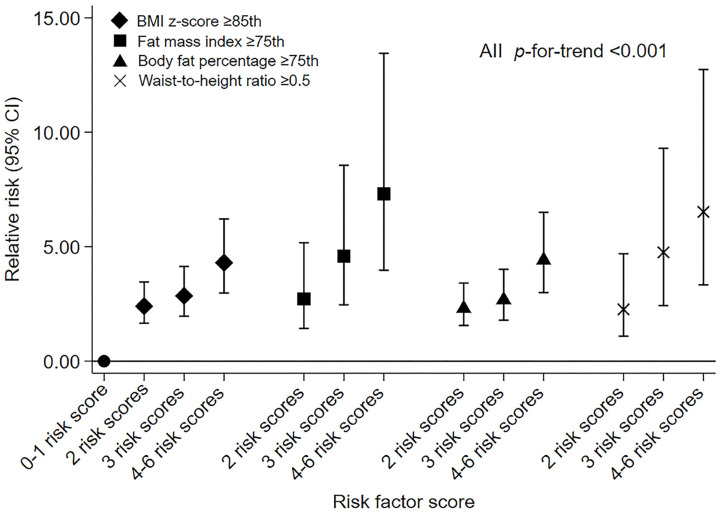
Associations of the number of risk factor scores with overweight/obesity risks in children aged 11–12 years (n = 942). All modes were adjusted for maternal age at conception, child’s sex, age at measurement, and birth weight. “●” indicated the baseline reference for the four outcomes. BMI: body mass index.

In the unadjusted and adjusted models, we all observed that children with higher risk scores had an increased risk of overweight/obesity (*p*-for-trend < 0.001 for all). In the adjusted model 1, compared with those with 0–1 risk scores, the children with 4–6 risk scores had 4.30 (95%CI: 2.98, 6.21) times higher risk of being overweight/obese; the RR for high FMI, BF%, and WHtR were 7.31 (3.97, 13.45), 4.41 (3.00, 6.50), and 6.52 (3.33, 12.74), respectively (Table 2 and Figure 2); a similar magnitude of results was observed in the adjusted model 2 (Table 2).

We further examined if there are interactions between two postnatal risk factors with the risk of overweight/obesity in children. Maternal overweight/obesity and breastfeeding duration < 6 months had addictive and multiplicative interactions on the risk of childhood overweight/obesity (Table S6), with all additive interaction indices (AP 0.39, 95%CI: 0.17–0.56; RERI 1.00, 95%CI: 0.41–1.56; SI 2.74, 95%CI: 1.12–6.72) and multiplicative interaction (RR: 2.37, 95%CI: 1.33–4.22) were statistically significant in adjusted model 1. These associations remained significant in the unadjusted model and adjusted model 3, which adjusted for some common prenatal factors. Tables S7–S9 show significant multiplicative interactions between maternal overweight/obesity and paternal overweight/obesity, paternal overweight/obesity and low family SES, and maternal overweight/obesity and tobacco exposure on the risk of childhood overweight/obesity, respectively.

The comparison between the imputed and available data set for the analytic sample is presented in Table S10. Sensitivity analyses showed similar associations of the postnatal risk factor scores with overweight/obesity risks in late childhood after using multiple imputations (Table S11 and Figure S1).

**Figure 3:**
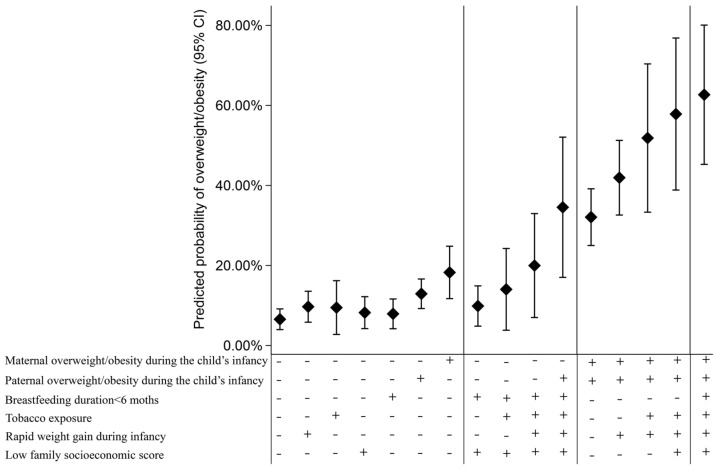
The adjusted predicted probability of overweight/obesity at 11–12 years according to different postnatal risk factor combinations (n = 942). “◆” indicates the adjusted predicted probability value. “+” indicates presence of risk factor, “−” indicates absence of risk factor. Bars show 95% confidence limits. This picture shows the adjusted predicted probability of overweight/obesity for each postnatal risk factor, as well as the minimum and maximum combinations of exposure to 2, 3, 4, 5, and 6 postnatal risk factors. The predicted probabilities are adjusted for maternal age at conception, child’s sex, age at measurement, and birth weight.

Table S5 shows the adjusted predicted probability of childhood overweight/obesity for all 64 combinations of the six postnatal risk factors: the probabilities ranged from 6.6% (4.0, 9.2) (with no risk factors) to 62.7% (45.3, 80.1) (with all six risk factors). Figure 3 shows the adjusted predicted probability of a child with overweight/obesity for each postnatal risk factor, as well as the minimum and maximum combinations of exposure to two, three, four, five, and six postnatal risk factors. For every single risk factor, the adjusted probability of overweight/obesity at 11–12 years ranged from 7.9% (4.2, 11.6) to 18.3% (11.7, 24.8). Maternal or paternal overweight/obesity accounted for 18.3% (11.7, 24.8) and 12.9% (9.2, 16.6), respectively, which contributed most to the adjusted predicted probabilities of childhood overweight/obesity, compared with other individual risk factors (Figure 3).

## 4. Discussion

In this longitudinal study, we observed that maternal and paternal overweight/obesity during the child’s infancy, tobacco exposure, breastfeeding duration < 6 months, RWG, during infancy, and low family SES were individually and significantly associated with four overweight/obesity indices. On analyzing the cumulative risks of these factors, which we aggregated into risk factor scores, we found that children with a postnatal risk score of 4 or more had a more than four-fold higher risk of being overweight/obese compared with children with a postnatal risk score of 0–1. And these cumulative risks were not influenced by the further adjustment of prenatal risk factors. In analyzing the interactive risk effects of these factors, we found that maternal overweight/obesity and breastfeeding duration < 6 months showed positive additive and multiplicative interactions on the risk of childhood overweight/obesity.

Our study highlights the postnatal period (children from birth to the age two) as an important window for childhood overweight/obesity prevention [35]. According to the Developmental Origins of Health and Disease hypothesis, exposure from birth to two years of age also affects the risk of future obesity risk [36]. More studies have examined risk factors exposed before the child’s birth than postnatal exposure [37,38,39]. Our study adjusted many typical prenatal factors that have been shown in other studies to strongly influence the risk of childhood overweight/obesity (such as maternal gestational diabetes [40,41], cesarean delivery [42,43], and macrosomia [44,45,46], small for gestational age [47].), and we still found the significant effect of postnatal risk factors on the risk of childhood obesity.

Some evidence showed that short breastfeeding duration [48,49,50], RWG [51,52], and lower family SES [53,54,55] are risk factors for childhood obesity, and our findings are in line with the literature. The relationship between tobacco exposure and childhood obesity, particularly postnatal exposure, is less commonly studied but we found that children were nearly 1.5 times more likely to be overweight/obese in childhood if someone smoked in the family. This was consistent with German research; tobacco exposure in children’s first year of life was positively associated with being overweight at age 6 years [OR: 2.94 (95% CI: 1.30, 6.67)] [56]. We further adjusted for maternal smoking during pregnancy, and the findings did not change. This indicates that infants may be more vulnerable to environmental tobacco exposure in the postnatal period. We did not find a significant association between the early introduction of solid foods < 4 months and the risk of childhood overweight/obesity, and there were mixed findings in the timing of solid foods introduction and childhood overweight/obesity [57]. It is noteworthy that parental overweight/obesity during the child’s infancy showed the largest effect on the risk of childhood overweight/obesity in our study, which is in line with the Growing Up in Singapore Towards Healthy Outcomes study [39]; children exposed to maternal or paternal overweight/obesity had the largest adjusted predicted probability of overweight/obesity (18.3% and 12.9%, respectively) at aged 11–12 years in our study. It is reported that the obesity-related behaviors of overweight/obese parents could influence the physiologic programming of the infant or children’s early life behaviors, leading to unhealthy weight trajectories among children [58,59]. This finding aligns with the concept of intergenerational transmission of obesity risk, emphasizing the need for family-centered prevention strategies.

Our results are consistent with an accumulation model with risk clustering, which suggests that the accumulation of different types of exposures (such as familial, socioeconomic, and behavioral) may cause long-term damage [60]. Those with more risk factors are more likely to become obese in late childhood. The adjusted predicted probability of being overweight/obese at 11–12 years ranged from 6.6% (with no risk factors) to 62.7% (with all risk factors). In our study, about half of children have 2–3 risk factors. For example, if a child has three risk factors, such as both parental overweight/obesity and tobacco exposure, the likelihood of being overweight/obese will increase to 41%, compared to 6.6% in those without risk factors. Therefore, we should take action to minimize postnatal risk factors to reduce the probability of childhood obesity.

In the interaction tests, we found additive and multiplicative interaction effects of maternal overweight/obesity during the child’s infancy and breastfeeding duration < 6 months, and multiplicative interactions between maternal overweight/obesity and paternal overweight/obesity during the child’s infancy, low family SES, and tobacco exposure. This means when offspring are exposed to multiple postnatal risk factors early in life, their risk of being overweight/obese in late childhood would be higher than a single exposure. This informs prevention effects should be applied through multiple levels during infancy.

Our findings highlight the pivotal role of simultaneous intervention in multiple postnatal risk factors in preventing future risk of overweight/obesity. On one hand, from birth to the age two would be a vital window, when behaviors are modifiable and physiologic characteristics are plastic [38]. A recent systematic review found that 60% of interventions targeting childhood obesity prevention in the first 1000 days ended when children were born [12], suggesting a missed opportunity to intervene in obesity during infancy. On the other hand, most intervention studies only address early risk factors individually rather than in combination, with limited potential beneficial effects on lowering children’s risk of overweight/obesity [61,62,63].

Our study has several strengths. Using a longitudinal study design with a large sample size and over a decade of follow-up allows us to conclude the long-term adverse effects of postnatal risk factors on the risk of childhood overweight/obesity. Postnatal life risk factors investigated in this study are representative. Our study also measured multiple overweight/obesity indices, including BMI, FMI, BF%, and WHtR, to comprehensively estimate the associations, which showed similar results to support the robustness of our findings.

Several limitations warrant further consideration. First, there is an under-representation of very disadvantaged families due to selective uptake of CheckPoint and attrition in LSAC. We conducted multiple imputations and performed a sensitivity analysis, and similar results were seen. Second, when collecting data on breastfeeding duration, we did not distinguish between exclusive and non-exclusive breastfeeding, which might lead to residual bias. Third, several exposures, such as parents’ BMI, and breastfeeding duration, were self-reported, which might lead to recall bias. Nevertheless, evidence suggests self-reported BMI correlates highly with actual measurements in adults, which is acceptable for longitudinal epidemiologic research [64]; and self-reported data on breastfeeding duration was valid and reliable, when recalled within three years [65]. Although our study provides insights into various postnatal risk factors for childhood obesity, other unmeasured risk factors, such as sleep patterns during infancy, deserve further exploration of their cumulative effects on childhood obesity in future studies.

## 5. Conclusions

Our study confirms that multiple modifiable postnatal risk factors including maternal and paternal overweight/obesity during the child’s infancy, tobacco exposure, low family SES, breastfeeding duration < 6 months, and RWG during infancy, significantly associated with the risk of childhood overweight/obesity, of which parental overweight/obesity during the child’s infancy impacted the most. The risk of childhood overweight/obesity increases with the number of risk factors. These findings underscore the critical nature of the postnatal period, from birth to the age two, as an intervention opportunity to lower the risk of being overweight/obesity later in childhood. Based on the evidence, we conclude that simultaneous intervention in various modifiable postnatal risk factors could be a more effective strategy for preventing obesity in children.

## Figures and Tables

**Table 1:** Characteristics of study participants.

Characteristics	Total (n = 1869)
Mothers	
Age (years)	31.4 ± 4.8 ^a^
Maternal pregnancy age (n, %)	
≥35 years	464 (25.3)
<35 years	1367 (74.7)
Maternal BMI during the child’s infancy (kg/m^2^)	25.2 ± 5.1
Maternal overweight/obesity during the child’s infancy ^b^ (n, %)	
BMI ≥ 25.0 kg/m^2^	948 (58.6)
BMI < 25.0 kg/m^2^	669 (41.4)
Gestational diabetes (n, %)	
Yes	95 (5.6)
No	1601 (94.4)
Maternal smoking during pregnancy (n, %)	
Yes	202 (11.9)
No	1501 (88.1)
Fathers	
Paternal BMI during the child’s infancy (kg/m^2^)	26.8 ± 3.8
Paternal overweight/obesity during the child’s infancy (n, %)	
BMI ≥ 25.0 kg/m^2^	488 (33.3)
BMI < 25.0 kg/m^2^	976 (66.7)
Children	
Sex (girls, %)	919 (49.0)
Gestational age (weeks)	39.2 ± 2.0
Age at CheckPoint (years)	11.5 ± 0.5
Birth weight (grams)	3443.9 ± 570.0
Delivered by cesarean section (n, %)	
Yes	560 (29.9)
No	1313 (70.1)
Child with assisted reproductive technology (n, %)	
Yes	135 (7.2)
No	1737 (92.8)
Duration of breastfeeding (n, %)	
≥6 months	1059 (64.0)
<6 months	595 (36.0)
Time of solid foods introduction (n, %)	
≥4 months	1605 (92.3)
<4 months	134 (7.7)
Tobacco exposure during infancy (n, %)	
Yes	107 (6.2)
No	1612 (93.8)
Increment of weight z-score during infancy	0.4 ± 0.5
BMI at 11–12 years (kg/m^2^)	19.2 ± 3.4
FMI at 11–12 years (kg/m^2^)	4.4 ± 2.6
BF% at 11–12 years (%)	21.7 ± 8.4
WHtR at 11–12 years	0.4 ± 0.1
Family socioeconomic score	0.3 ± 1.0

^a^ Mean ± SD or n (%); ^b^ BMI category was based on the WHO’s criteria: BMI < 25.0 kg/m^2^ Normal and underweight; BMI ≥ 25.0 kg/m^2^ as overweight and obese. BMI: body mass index; FMI: fat mass index; BF%: body fat percentage; WHtR: waist-to-height ratio.

**Table 2:** Associations of the number of postnatal risk factor scores with overweight/obesity risks in 11–12-year-old children (n = 942).

	Unadjusted Model		Adjusted Model 1		Adjusted Model 2	
Number of Risk Factors	RR (95%CI)	*p*-Value	RR (95%CI)	*p*-Value	RR (95%CI)	*p*-Value
Primary Outcome for Children						
Overweight or obesity ^a^						
2 risk scores	2.34 (1.62 to 3.39)	<0.001	2.40 (1.66 to 3.46)	<0.001	2.49 (1.70 to 3.64)	<0.001
3 risk scores	2.77 (1.90 to 4.05)	<0.001	2.85 (1.96 to 4.14)	<0.001	2.89 (1.95 to 4.29)	<0.001
≥4 risk scores	3.92 (2.70 to 5.68)	<0.001	4.30 (2.98 to 6.21)	<0.001	4.32 (2.91 to 6.41)	<0.001
*p*-for-trend	<0.001		<0.001		<0.001	
Secondary Outcomes for Children						
Fat mass index (≥75th) ^b^						
2 risk scores	2.64 (1.39 to 5.04)	<0.01	2.72 (1.43 to 5.17)	<0.01	2.69 (1.39 to 5.21)	<0.01
3 risk scores	4.45 (2.38 to 8.31)	<0.001	4.59 (2.46 to 8.55)	<0.001	4.10 (2.11 to 7.98)	<0.001
≥4 risk scores	6.69 (3.61 to 12.39)	<0.001	7.31 (3.97 to 13.45)	<0.001	6.59 (3.42 to 12.69)	<0.001
*p*-for-trend	<0.001		<0.001		<0.001	
Body fat percentage (≥75th) ^b^						
2 risk scores	2.25 (1.52 to 3.32)	<0.001	2.30 (1.56 to 3.41)	<0.001	2.29 (1.54 to 3.40)	<0.001
3 risk scores	2.61 (1.74 to 3.91)	<0.001	2.68 (1.79 to 4.01)	<0.001	2.43 (1.59 to 3.70)	<0.001
≥4 risk scores	4.09 (2.78 to 6.03)	<0.001	4.41 (3.00 to 6.50)	<0.001	4.19 (2.78 to 6.30)	<0.001
*p*-for-trend	<0.001		<0.001		<0.001	
Waist-to-height ratio (≥0.5)						
2 risk scores	2.18 (1.05 to 4.53)	0.04	2.26 (1.09 to 4.69)	0.03	2.19 (1.03 to 4.65)	0.05
3 risk scores	4.73 (2.40 to 9.32)	<0.001	4.75 (2.43 to 9.30)	<0.001	4.00 (1.97 to 8.13)	<0.001
≥4 risk scores	6.05 (3.04 to 12.03)	<0.001	6.52 (3.33 to 12.74)	<0.001	5.23 (2.53 to 10.80)	<0.001
*p*-for-trend	<0.001		<0.001		<0.001	

All effect estimates are referenced to children with 0–1 risk factor (n = 354). Six risk factors are maternal and paternal overweight/obesity during the child’s infancy, low family socioeconomic score, breastfeeding duration < 6 months, rapid weight gain during infancy, and tobacco exposure. ^a^ Age- and sex-specific body mass index (BMI) z scores were calculated using the Centers for Disease Control and Prevention sex-specific BMI-for-age growth charts from 2000, having a healthy weight was defined as having a BMI z score < 85th percentile, and having overweight/obesity was defined as having a BMI z scores ≥ 85th percentile for age and sex. ^b^ Participants with a body fat percentage (BF%) and/or fat mass index (FMI) greater than or equal to the age- and gender-specific 75th percentile was classified as overweight/obese, according to published reference Curves for American National Health and Nutrition Examination Survey (NHANES) IV for BF% and FMI. Adjusted model 1: adjusted for maternal age at conception, child’s sex, age at measurement, and birth weight. Adjusted model 2: model 1 + adjusted for the child’s gestational age, delivery by cesarean section (yes or no), assisted reproductive technology for conception (yes or no), time of solid foods introduction (≥4 months or <4 months), maternal smoking during pregnancy (yes or no), and maternal gestational diabetes (yes or no). RR: relative risk. CI: confidence interval.

## Data Availability

Dataset and technical documents are available from Growing Up in Australia: The Longitudinal Study of Australian Children via a low-cost license for bone fide researchers. More information is available at https://www.growingupinaustralia.gov.au (accessed on 3 April 2024).

## Supplementary Materials

The following supporting information can be downloaded at https://www.mdpi.com/article/10.3390/nu16101536/s1, Table S1: Information of early life risk factors collected at LSAC wave 1; Table S2. Univariate logistic regression analysis of seven postnatal risk factors and risk of overweight/obesity in children aged 11–12 years; Table S3. Characteristics of study participants according to the postnatal risk factor scores; Table S4. Comparison of characteristics between included and excluded participants of accumulative risk analyses; Table S5. The adjusted predicted probability of overweight/obese in children aged 11–12 years for all 64 combinations of six postnatal risk factors; Table S6. Comparison of characteristics between the imputed and analytic data; Table S7. Interaction effect of maternal overweight/obesity and children’s breastfeeding duration < 6 months on the risk of overweight/obesity in late childhood; Table S8. Interaction effect of maternal overweight/obesity and paternal overweight/obesity on the risk of overweight/obesity in late childhood; Table S9. Interaction effect of paternal overweight/obesity and low family socioeconomic score on the risk of overweight/obesity in late childhood; Table S10. Interaction effect of maternal overweight/obesity and tobacco exposure on the risk of overweight/obesity in late childhood; Table S11. Associations of the postnatal risk factor scores with overweight/obesity in children aged 11–12 years: finding from imputed data (n = 1874); Figure S1. Associations of the postnatal risk factor scores with overweight/obesity risks in children aged 11–12 years: findings from imputed data (n = 1874).

## References

[1] NCD Risk Factor Collaboration (NCD-RisC) (2017). Worldwide trends in body-mass index, underweight, overweight, and obesity from 1975 to 2016: A pooled analysis of 2416 population-based measurement studies in 128·9 million children, adolescents, and adults. Lancet.

[2] World Health Organization Consideration of the Evidence on Childhood Obesity for the Commission on Ending Childhood Obesity: Report of the ad hoc Working Group on Science and Evidence for Ending Childhood Obesity, Geneva, Switzerland. https://apps.who.int/iris/handle/10665/206549.

[3] Blake-Lamb T.L., Locks L.M., Perkins M.E., Woo Baidal J.A., Cheng E.R., Taveras E.M. (2016). Interventions for childhood obesity in the first 1,000 days a systematic review. Am. J. Prev. Med..

[4] Godfrey K.M., Reynolds R.M., Prescott S.L., Nyirenda M., Jaddoe V.W., Eriksson J.G., Broekman B.F. (2017). Influence of maternal obesity on the long-term health of offspring. Lancet Diabetes Endocrinol..

[5] Diesel J.C., Eckhardt C.L., Day N.L., Brooks M.M., Arslanian S.A., Bodnar L.M. (2015). Is gestational weight gain associated with offspring obesity at 36 months?. Pediatr. Obes..

[6] Aris I.M., Soh S.E., Tint M.T., Saw S.M., Rajadurai V.S., Godfrey K.M., Gluckman P.D., Yap F., Chong Y.S., Lee Y.S. (2015). Associations of gestational glycemia and prepregnancy adiposity with offspring growth and adiposity in an Asian population. Am. J. Clin. Nutr..

[7] Hou W., Zhang M., Ji Y., Hong X., Wang G., Xu R., Liang L., Saria S., Ji H. (2022). A prospective birth cohort study of maternal prenatal cigarette smoking assessed by self-report and biomarkers on childhood risk of overweight or obesity. Precis. Nutr..

[8] Liao Z., Wang J., Chen F., Chen Y., Zhang T., Liu G., Xie X., Tai J. (2022). Association of cesarean delivery with trajectories of growth and body composition in preschool children. Nutrients.

[9] Zheng M., Campbell K.J., Baur L., Rissel C., Wen L.M. (2021). Infant feeding and growth trajectories in early childhood: The application and comparison of two longitudinal modelling approaches. Int. J. Obes..

[10] Zhou J., Dang S., Zeng L., Gao W., Wang D., Li Q., Jiang W., Pei L., Li C., Yan H. (2016). Rapid infancy weight gain and 7- to 9-year childhood obesity risk: A prospective cohort study in rural western China. Medicine.

[11] Lioret S., Harrar F., Boccia D., Hesketh K.D., Kuswara K., Van Baaren C., Maritano S., Charles M.A., Heude B., Laws R. (2023). The effectiveness of interventions during the first 1000 days to improve energy balance-related behaviors or prevent overweight/obesity in children from socio-economically disadvantaged families of high-income countries: A systematic review. Obes. Rev..

[12] Pérez-Muñoz C., Carretero-Bravo J., Ortega-Martín E., Ramos-Fiol B., Ferriz-Mas B., Díaz-Rodríguez M. (2022). Interventions in the first 1000 days to prevent childhood obesity: A systematic review and quantitative content analysis. BMC Public Health.

[13] Gillman M.W., Ludwig D.S. (2013). How early should obesity prevention start?. N. Engl. J. Med..

[14] Sanson A.V., Johnstone R.E. (2004). Growing Up in Australia takes its first steps. Fam. Matters.

[15] Soloff C., Lawrence D., Johnstone R. (2005). Sample Design.

[16] Clifford S.A., Davies S., Wake M. (2019). Child Health CheckPoint: Cohort summary and methodology of a physical health and biospecimen module for the Longitudinal Study of Australian Children. BMJ Open.

[17] Wake M., Clifford S., York E., Mensah F., Gold L., Burgner D., Davies S. (2014). Introducing Growing Up in Australia’s Child Health CheckPoint. Fam. Matters.

[18] World Health Organization (2000). Obesity: Preventing and Managing the Global Epidemic.

[19] Section on Breastfeeding (2012). Breastfeeding and the use of human milk. Pediatrics.

[20] Kuczmarski R.J., Ogden C.L., Grummer-Strawn L.M., Flegal K.M., Guo S.S., Wei R., Mei Z., Curtin L.R., Roche A.F., Johnson C.L. (2000). CDC growth charts: United States. Adv. Data.

[21] Monteiro P.O., Victora C.G. (2005). Rapid growth in infancy and childhood and obesity in later life—A systematic review. Obes. Rev..

[22] Druet C., Stettler N., Sharp S., Simmons R.K., Cooper C., Smith G.D., Ekelund U., Lévy-Marchal C., Jarvelin M.R., Kuh D. (2012). Prediction of childhood obesity by infancy weight gain: An individual-level meta-analysis. Paediatr. Perinat. Epidemiol..

[23] Taveras E.M., Rifas-Shiman S.L., Sherry B., Oken E., Haines J., Kleinman K., Rich-Edwards J.W., Gillman M.W. (2011). Crossing growth percentiles in infancy and risk of obesity in childhood. Arch. Pediatr. Adolesc. Med..

[24] Baker K., Sipthorp M., Edwards B. (2017). A Longitudinal Measure of Socioeconomic Position in LSAC.

[25] Huh S.Y., Rifas-Shiman S.L., Taveras E.M., Oken E., Gillman M.W. (2011). Timing of solid food introduction and risk of obesity in preschool-aged children. Pediatrics.

[26] Gingras V., Aris I.M., Rifas-Shiman S.L., Switkowski K.M., Oken E., Hivert M.F. (2019). Timing of complementary feeding introduction and adiposity throughout childhood. Pediatrics.

[27] AIoF S. (2015). Longitudinal Study of Australian Children Data User Guide–November 2015.

[28] Clifford S.A., Gillespie A.N., Olds T., Grobler A.C., Wake M. (2019). Body composition: Population epidemiology and concordance in Australian children aged 11–12 years and their parents. BMJ Open.

[29] Weber D.R., Moore R.H., Leonard M.B., Zemel B.S. (2013). Fat and lean BMI reference curves in children and adolescents and their utility in identifying excess adiposity compared with BMI and percentage body fat. Am. J. Clin. Nutr..

[30] Laurson K.R., Eisenmann J.C., Welk G.J. (2011). Body fat percentile curves for U.S. children and adolescents. Am. J. Prev. Med..

[31] McCarthy H.D., Ashwell M. (2006). A study of central fatness using waist-to-height ratios in UK children and adolescents over two decades supports the simple message—‘keep your waist circumference to less than half your height’. Int. J. Obes..

[32] Sharma A.K., Metzger D.L., Daymont C., Hadjiyannakis S., Rodd C.J. (2015). LMS tables for waist-circumference and waist-height ratio z-scores in children aged 5–19 y in NHANES III: Association with cardio-metabolic risks. Pediatr. Res..

[33] Zou G. (2004). A modified Poisson regression approach to prospective studies with binary data. Am. J. Epidemiol..

[34] Jang Y.J., Kang C., Myung W., Lim S.W., Moon Y.K., Kim H., Kim D.K. (2021). Additive interaction of mid- to late-life depression and cerebrovascular disease on the risk of dementia: A nationwide population-based cohort study. Alzheimers Res. Ther..

[35] Marousez L., Lesage J., Eberlé D. (2019). Epigenetics: Linking early postnatal nutrition to obesity programming?. Nutrients.

[36] Gillman M.W. (2005). Developmental origins of health and disease. N. Engl. J. Med..

[37] Hu J., Aris I.M., Lin P.D., Rifas-Shiman S.L., Perng W., Woo Baidal J.A., Wen D., Oken E. (2021). Longitudinal associations of modifiable risk factors in the first 1000 days with weight status and metabolic risk in early adolescence. Am. J. Clin. Nutr..

[38] Gillman M.W., Rifas-Shiman S.L., Kleinman K., Oken E., Rich-Edwards J.W., Taveras E.M. (2008). Developmental origins of childhood overweight: Potential public health impact. Obesity.

[39] Aris I.M., Bernard J.Y., Chen L.W., Tint M.T., Pang W.W., Soh S.E., Saw S.M., Shek L.P., Godfrey K.M., Gluckman P.D. (2018). Modifiable risk factors in the first 1000 days for subsequent risk of childhood overweight in an Asian cohort: Significance of parental overweight status. Int. J. Obes..

[40] Zhu B.B., Gao H., Geng M.L., Wu X., Tong J., Deng F., Zhang S.Y., Wu L.H., Huang K., Wu X.Y. (2022). Sex discrepancy observed for gestational metabolic syndrome parameters and polygenic risk associated with preschoolers’ BMI growth trajectory: The Ma’anshan birth cohort study. Front. Endocrinol..

[41] Zhao P., Liu E., Qiao Y., Katzmarzyk P.T., Chaput J.P., Fogelholm M., Johnson W.D., Kuriyan R., Kurpad A., Lambert E.V. (2016). Maternal gestational diabetes and childhood obesity at age 9–11: Results of a multinational study. Diabetologia.

[42] Sitarik A.R., Havstad S.L., Johnson C.C., Jones K., Levin A.M., Lynch S.V., Ownby D.R., Rundle A.G., Straughen J.K., Wegienka G. (2020). Association between cesarean delivery types and obesity in preadolescence. Int. J. Obes..

[43] Kuhle S., Tong O.S., Woolcott C.G. (2015). Association between caesarean section and childhood obesity: A systematic review and meta-analysis. Obes. Rev..

[44] Zheng M., Hesketh K.D., Vuillermin P., Dodd J., Wen L.M., Baur L.A., Taylor R., Byrne R., Mihrshahi S., Burgner D. (2023). Understanding the pathways between prenatal and postnatal factors and overweight outcomes in early childhood: A pooled analysis of seven cohorts. Int. J. Obes..

[45] Weng S.F., Redsell S.A., Swift J.A., Yang M., Glazebrook C.P. (2012). Systematic review and meta-analyses of risk factors for childhood overweight identifiable during infancy. Arch. Dis. Child..

[46] Yang Z., Dong B., Song Y., Wang X., Dong Y., Gao D., Li Y., Zou Z., Ma J., Arnold L. (2020). Association between birth weight and risk of abdominal obesity in children and adolescents: A school-based epidemiology survey in China. BMC Public Health.

[47] Tur J.A., Martinez J.A. (2022). Guide and advances on childhood obesity determinants: Setting the research agenda. Obes. Rev..

[48] Qiao J., Dai L.J., Zhang Q., Ouyang Y.Q. (2020). A meta-analysis of the association between breastfeeding and early childhood obesity. J. Pediatr. Nurs..

[49] Huang H., Gao Y., Zhu N., Yuan G., Li X., Feng Y., Gao L., Yu J. (2022). The effects of breastfeeding for four months on thinness, overweight, and obesity in children aged 3 to 6 years: A retrospective cohort study from national physical fitness surveillance of Jiangsu province, China. Nutrients.

[50] Yan J., Liu L., Zhu Y., Huang G., Wang P.P. (2014). The association between breastfeeding and childhood obesity: A meta-analysis. BMC Public Health.

[51] Lindholm A., Almquist-Tangen G., Alm B., Bremander A., Dahlgren J., Roswall J., Staland-Nyman C., Bergman S. (2022). Early rapid weight gain, parental body mass index and the association with an increased waist-to-height ratio at 5 years of age. PLoS ONE.

[52] Lin Q., Jiang Y., Wang G., Sun W., Dong S., Deng Y., Meng M., Zhu Q., Mei H., Zhou Y. (2021). Combined effects of weight change trajectories and eating behaviors on childhood adiposity status: A birth cohort study. Appetite.

[53] Hoebel J., Waldhauer J., Blume M., Schienkiewitz A. (2022). Socioeconomic status, overweight, and obesity in childhood and adolescence—Secular trends from the nationwide German kiggs study. Dtsch. Arztebl. Int..

[54] Costa-Font J., Gil J. (2013). Intergenerational and socioeconomic gradients of child obesity. Soc. Sci. Med..

[55] Ke Y., Zhang S., Hao Y., Liu Y. (2023). Associations between socioeconomic status and risk of obesity and overweight among Chinese children and adolescents. BMC Public Health.

[56] Raum E., Küpper-Nybelen J., Lamerz A., Hebebrand J., Herpertz-Dahlmann B., Brenner H. (2011). Tobacco smoke exposure before, during, and after pregnancy and risk of overweight at age 6. Obesity.

[57] Pearce J., Taylor M.A., Langley-Evans S.C. (2013). Timing of the introduction of complementary feeding and risk of childhood obesity: A systematic review. Int. J. Obes..

[58] Cole T.J., Power C., Moore G.E. (2008). Intergenerational obesity involves both the father and the mother. Am. J. Clin. Nutr..

[59] Nader P.R., Huang T.T., Gahagan S., Kumanyika S., Hammond R.A., Christoffel K.K. (2012). Next steps in obesity prevention: Altering early life systems to support healthy parents, infants, and toddlers. Child. Obes..

[60] Kuh D., Ben-Shlomo Y., Lynch J., Hallqvist J., Power C. (2003). Life course epidemiology. J. Epidemiol. Community Health.

[61] Hoffmann J., Günther J., Stecher L., Spies M., Meyer D., Kunath J., Raab R., Rauh K., Hauner H. (2019). Effects of a lifestyle intervention in routine care on short- and long-term maternal weight retention and breastfeeding behavior-12 months follow-up of the Cluster-Randomized GeliS Trial. J. Clin. Med..

[62] Louzada M.L., Campagnolo P.D., Rauber F., Vitolo M.R. (2012). Long-term effectiveness of maternal dietary counseling in a low-income population: A randomized field trial. Pediatrics.

[63] Helle C., Hillesund E.R., Wills A.K., Øverby N.C. (2019). Evaluation of an eHealth intervention aiming to promote healthy food habits from infancy -the Norwegian randomized controlled trial Early Food for Future Health. Int. J. Behav. Nutr. Phys. Act..

[64] Wright F.L., Green J., Reeves G., Beral V., Cairns B.J. (2015). Validity over time of self-reported anthropometric variables during follow-up of a large cohort of UK women. BMC Med. Res. Methodol..

[65] Li R., Scanlon K.S., Serdula M.K. (2005). The validity and reliability of maternal recall of breastfeeding practice. Nutr. Rev..

