# Supplementary material for: The Accumulative Effect of Multiple Postnatal Risk Factors with the Risk of Being Overweight/Obese in Late Childhood
Source: Nutrients. 2024 May 20;16(10):1536. doi: 10.3390/nu16101536 (PMC11124345; doi:10.3390/nu16101536)
Supplement: Supplementary file 1 [file nutrients-16-01536-s001.zip › nutrients-2980099-supplementary.pdf]

Supplemental Materials

The Accumulative Effect of Multiple Postnatal Risk Factors with the Risk of being Overweight/Obese in Late Childhood

Ting Wu [1,2,†], Zijun Liao [3,†], Jing Wang [4,5] and Mengjiao Liu [1,2,*]

School of Public Health, Jiangxi Medical College, Nanchang University, Nanchang 330006, China; wuting@email.ncu.edu.cn

2 Jiangxi Provincial Key Laboratory of Disease Prevention and Public Health, Nanchang University, Nanchang 330006, China

3 Capital Institute of Pediatrics, Beijing 100020, China; liaozijun43@163.com

4 Murdoch Children's Research Institute, The Royal Children's Hospital, Parkville, VIC 3052, Australia; jing.wang@mcri.edu.au

5 Department of Pediatrics, The University of Melbourne, Parkville, VIC 3010, Australia

* Correspondence: liumengjiao@ncu.edu.cn; Tel.: +86-0791-8635-3070

† These authors contributed equally to this work.

Contact info:

Mengjiao Liu; School of Public Health, Jiangxi Medical College, Nanchang University, Nanchang 330006, China; liumengjiao@ncu.edu.cn

Table S1. Information of early life risk factors collected at LSAC wave 1.
Table S2. Modified Poisson regression analysis of seven postnatal risk factors and risk of overweight/obesity in children aged 11-12 years.
Table S3. Characteristics of study participants according to the postnatal risk factor scores.
Table S4. Comparison of characteristics between included and excluded participants of accumulative risk analyses.
Table S5. The adjusted predicted probability of overweight/obese in children aged 11-12 years for all 64 combinations of six postnatal risk factors.
Table S6. Interaction effect of maternal overweight/obesity during the child's infancy and children's breastfeeding duration < 6 months on the risk of overweight/obesity in late childhood.
Table S7. Interaction effect of maternal overweight/obesity and paternal overweight/obesity during the child's infancy on the risk of overweight/obesity in late childhood.
Table S8. Interaction effect of paternal overweight/obesity during the child's infancy and low family socioeconomic score on the risk of overweight/obesity in late childhood.
Table S9. Interaction effect of maternal overweight/obesity during the child's infancy and tobacco exposure on the risk of overweight/obesity in late childhood.

Table S10. Comparison of characteristics between the imputed and analytic data.
Table S11. Associations of the postnatal risk factor scores with overweight/obesity in children aged 11-12 years: finding from imputed data (n = 1874).
Figure S1. Associations of the postnatal risk factor scores with overweight/obesity risks in children aged 11-12 years: findings from imputed data (n = 1874).

Table S1. Information of early life risk factors collected at LSAC wave 1.

Exposure Measures	Brief Protocol
Maternal BMI during the child's infancy	Mother's height and weight were self-reported, BMI = weight (kg)/ height (m^2)
Paternal BMI during the child's infancy	Father's height and weight were self-reported, BMI = weight (kg)/ height (m^2)
Maternal pregnancy age	The age of the mother at conception was calculated based on the age reported by the mother in wave 1 and the age of the child.
Maternal gestational diabetes	During this pregnancy, did you have diabetes? (Yes/No)
Birth weight	The interviewer copied these from the Parent Health Record recorded by the birthing hospital (the great majority). If the record was not available, interviewers asked parents "How much did the child weigh at birth? (Grams)"
Gestational age	The interviewer copied these from the Parent Health Record recorded by the birthing hospital (the great majority). If the record was not available, interviewers asked parents "After how many weeks of pregnancy was the child born?"
Delivery type	What type of birth, or delivery, was it caesarean? (Yes/ No)
Mode of conception	Were fertility drugs, in-vitro fertilization, or other medical treatments needed to get pregnant with this child? (Yes/No)
Breastfeeding Duration	How old was the child when he/she completely stopped being breastfed? (Include expressed breast milk) (Age in days)
Time of solid foods introduction	How old was the child when he/she first had solid food regularly? (Regularly = more than twice a week for several continuous weeks. Solid food = baby cereals, pureed fruits, etc. — not milk or drinks) (Age in days)
Increment of weight z-score during infancy	Infant weight at wave 1 was measured on a Salter Australia scale by weighing the infant with an adult, then the adult alone, and subtracting the latter from the former. LSAC computed age - and gender-specific weight-for-age z scores using the 2000 Centers for Disease Control and Prevention Growth Charts [20]. To calculate the change in the weight-for-age z score during infancy, we subtracted the weight-for-age z score at birth from the weight-for-age z score before the age of two.

Tobacco exposure	Including yourself, how many people who live with you smoke inside the house? (Number)
Maternal smoking during pregnancy	During the pregnancy with the study child, did you smoke cigarettes? (Yes/No)
Family socioeconomic score	The LSAC composite household socioeconomic status (SES) variable was constructed by Blakemore et al [24] and was derived from standardized scores for combined annual household income; parents' years of education; and parents' occupations. The summary measure of SES developed for each family produced a continuous score, with higher scores indicating better SES.

References:

20 Kuczmarski, R.J.; Ogden, C.L.; Grummer-Strawn, L.M.; Flegal, K.M.; Guo, S.S.; Wei, R.; Mei, Z.; Curtin, L.R.; Roche, A.F.; Johnson, C.L. CDC growth charts: United States. *Adv. Data* 2000, 314, 311–327

24 Baker, K.; Sipthorp, M.; Edwards, B. A Longitudinal Measure of Socioeconomic Position in LSAC; *Australian Institute of Family Studies: Canberra, Australian*, **2017**.

Table S2. Modified Poisson regression analysis of seven postnatal risk factors and risk of overweight/obesity in children aged 11-12 years.

	**Overweight/obese [a]**		**Fat mass index ≥ 75th[b]**		**Body fat % ≥ 75th[b]**		**Waist height to ratio ≥ 0.5**	
**Risk factor**	**RR (95%CI)**	***P-value***	**RR (95%CI)**	***P-value***	**RR (95%CI)**	***P-value***	**RR (95%CI)**	***P-value***
Maternal overweight/obesity during the child's infancy								
BMI < 25.0 kg/m²	1		1		1		1	
BMI ≥ 25.0 kg/m²	2.37 (1.97 to 2.87)	<.001	3.06 (2.24 to 4.17)	<.001	2.33 (1.91 to 2.84)	<.001	3.43 (2.41 to 4.89)	<.001
Paternal overweight/obesity during the child's infancy								
BMI < 25.0 kg/m²	1		1		1		1	
BMI ≥ 25.0 kg/m²	1.77 (1.39 to 2.25)	<.001	2.18 (1.45 to 3.29)	<.001	1.62 (1.26 to 2.08)	<.001	3.07 (1.83 to 5.14)	<.001
Breastfeeding duration < 6 months								
Yes	1		1		1		1	
No	1.24 (1.03 to 1.48)	.05	1.51 (1.14 to 1.99)	.01	1.23 (1.02 to 1.48)	.06	1.61 (1.18 to 2.20)	<.01
Family socioeconomic score								
≥ 25th	1		1		1		1	
< 25th	1.51 (1.27 to 1.79)	<.001	1.69 (1.29 to 2.22)	<.001	1.59 (1.33 to 1.89)	<.001	1.69 (1.25 to 2.29)	.001
Tobacco exposure								
No	1		1		1		1	
Yes	1.76 (1.36 to 2.29)	.001	2.25 (1.52 to 3.34)	<.001	1.97 (1.52 to 2.56)	<.001	2.11 (1.34 to 3.34)	.003
Time of solid foods introduction								
≥ 4 months	1		1		1		1	
< 4 months	1.16 (0.87 to 1.55)	.40	0.98 (0.59 to 1.64)	.95	1.11 (0.81 to 1.52)	.59	0.98 (0.56 to 1.71)	.94
Increment of weight z-score during infancy								
≤ 0.67	1		1		1		1	
> 0.67	1.54 (1.26 to 1.89)	<.001	1.64 (1.20 to 2.24)	.004	1.33 (1.08 to 1.63)	.02	1.61 (1.14 to 2.29)	.01

[a] Overweight/obese was defined: as having a body mass index z-score ≥ 85th percentile, according to the Centers for Disease Control and Prevention in the United States

[b] Participants with a body fat percentage and/or fat mass index greater than or equal to the age- and gender-specific 75th percentile was classified as overweight or obese, according to published reference Curves for American National Health and Nutrition Examination Survey (NHANES) IV for and fat mass index.
All models were adjusted for maternal age at conception, child's sex, age at measurement, and birth weight.

Table S3. Characteristics of study participants according to postnatal risk factor scores.

Characteristic	0 or 1 (n=354)	2 (n=273)	3 (n=187)	4 to 6 (n=128)
**Mothers**				
Age (years)	32.2 ± 4.1	32.3 ± 4.4	31.2 ± 4.8	30.3 ± 5.5
Maternal pregnancy age (n, %)				
≥ 35 years	98 (27.7)	83 (30.4)	46 (24.6)	26 (20.3)
< 35 years	256 (72.3)	190 (69.6)	141 (75.4)	102 (79.7)
Maternal BMI during the child's infancy (kg/m^2)	22.3 ± 2.7	24.7 ± 4.6	27.1 ± 4.8	29.9 ± 5.8
Maternal overweight/obesity during the child's infancy [b] (n, %)				
BMI ≥ 25.0 kg/m^2	29 (8.2)	113 (41.4)	59 (31.6)	22(17.2)
BMI < 25.0 kg/m^2	325 (91.8)	160 (58.6)	128 (68.4)	106 (81.8)
Gestational diabetes (n, %)				
Yes	12 (3.4)	18 (6.7)	10 (5.4)	8 (6.4)
No	340 (96.6)	252 (93.3)	175 (94.6)	116 (93.6)
**Fathers**				
Paternal BMI during the child's infancy (kg/m^2)	25.2 ± 3.2	27.0 ± 3.2	27.6 ± 3.8	29.1 ± 4.4
Paternal overweight/obesity during the child's infancy [b] (n, %)				
BMI ≥ 25.0 kg/m^2	156 (44.1)	74 (27.1)	40 (21.4)	11 (8.6)
BMI < 25.0 kg/m^2	198 (55.9)	199 (72.9)	147 (78.6)	117 (91.4)
**Children**				
Sex (girls, %)	182 (51.4)	145 (53.1)	83 (44.4)	63 (49.2)
Age (years)	11.57 ± 0.50	11.55 ± 0.51	11.60 ± 0.49	11.60 ± 0.49
Birth weight (grams)	3571.4 ± 487.0	3401.5 ± 566.9	3348.7 ± 599.6	3274.8 ± 582.5
Delivered by cesarean section (n, %)				
Yes	82 (23.2)	77 (28.2)	68 (36.4)	47 (37.0)
No	272 (76.8)	196 (71.8)	119 (63.6)	80 (63.0)
The child with assisted reproductive technology (n, %)				
Yes	24 (6.8)	18 (6.6)	18 (9.6)	12 (9.4)
No	330 (93.2)	254 (93.4)	169 (90.4)	116 (90.6)
Breastfeeding duration (n, %)				
≥ 6 months	334 (94.3)	203 (74.4)	79 (42.3)	12 (9.4)
< 6 months	20 (5.7)	70 (25.6)	108 (57.7)	116 (90.6)
Time of solid foods introduction (n, %)				
≥ 4 months	335 (96.0)	257 (95.2)	171 (92.4)	106 (85.5)
< 4 months	14 (4.0)	13 (4.8)	14 (7.6)	18 (14.5)
Tobacco exposure (n, %)				

Yes	2 (0.6)	8 (2.9)	13 (6.9)	21 (16.5)
No	352 (99.4)	265 (97.1)	174 (93.1)	106 (83.5)
Increment of weight z-score during infancy	0.1 ± 0.3	0.4 ± 0.5	0.6 ± 0.5	0.8 ± 0.4
BMI at 11-12 years (kg/m²)	18.2 ± 2.4	18.9 ± 3.0	19.7 ± 3.7	21.2 ± 4.5
FMI at 11-12 years (kg/m²)	3.6 ± 1.8	4.3 ± 2.3	4.7 ± 2.9	5.9 ± 3.5
BF% at 11-12 years (%)	19.1 ± 6.8	21.6 ± 8.3	22.6 ± 8.7	25.9 ± 10.0
WHtR at 11-12 years	0.4 ± 0.0	0.4 ± 0.1	0.4 ± 0.1	0.5 ± 0.1
Family socioeconomic score	0.8 ± 0.8	0.5 ± 0.9	0.2 ± 0.9	-0.4 ± 0.7

BMI: body mass index; FMI: fat mass index; BF%: body fat percentage; WHtR: waist-to-height ratio.

Table S4. Comparison of characteristics between included and excluded participants of accumulative risk analyses.

Characteristic	Excluded (N=932)	Included (N=942)	*P-value*
**Mothers**			
Age (years)	31.00 ± 5.09	31.74 ± 4.57	.001
BMI during the child's infancy (kg/m²)	25.49 ± 5.32	24.98 ± 4.96	.05
College graduate or higher	711 (76.4)	749 (79.6)	.09
Gestational diabetes (n, %)			.38
Yes	47 (6.1)	48 (5.2)	
No	718 (93.9)	883 (94.8)	
**Fathers**			
BMI during the child's infancy (kg/m²)	26.95 ± 3.95	26.73 ± 3.74	.28
**Child**			
Female	446 (47.9)	473 (50.2)	.31
Age (years)	11.43 ± 0.50	11.58 ± 0.50	<.001
Birthweight (grams)	3450.3 ± 584.3	3437.6 ± 555.9	.63
Delivered by cesarean section (n, %)			.46
Yes	286 (30.7)	274 (29.1)	
No	646 (69.3)	667 (70.9)	
The child with assisted reproductive technology (n, %)			.46
Yes	63 (6.8)	72 (7.7)	
No	868 (93.2)	869 (92.3)	
Breastfeeding duration (n, %)			.01
≥ 6 months	431 (60.5)	628 (66.7)	
< 6 months	281 (39.5)	314 (33.3)	
Time of solid foods introduction (n, %)			
≥ 4 months	736 (90.8)	869 (93.6)	.03
< 4 months	75 (9.2)	59 (6.4)	
Tobacco exposure (n, %)			<.01
Yes	63 (8.1)	44 (4.7)	
No	715 (91.9)	897 (95.3)	
Increment of weight z-score during infancy	0.59 ± 1.18	0.32 ± 1.19	<.001
BMI at 11-12 years (kg/m²)	19.33 ± 3.51	19.10 ± 3.35	.16
FMI at 11-12 years (kg/m²)	4.51 ± 2.64	4.33 ± 2.57	.13

BF% at 11-12 years (%)	22.04 ± 8.40	21.43 ± 8.41	.12
WHtR at 11-12 years	0.43 ± 0.05	0.43 ± 0.05	.01
Family socioeconomic score	0.21 ± 0.98	0.42 ± 0.91	<.001

BMI: body mass index; FMI: fat mass index; BF%: body fat percentage; WHtR: waist-to-height ratio.

Table S5. The adjusted predicted probability of overweight/obese in children aged 11-12 years for all 64 combinations of six postnatal risk factors.

Order	Combinations of risk factors	z	Predicted probability (%, 95% CI)	*P-value*
	**Population with 0 risk factor**			
1	No risk factor	4.98	6.56 (3.98 to 9.15)	<.001
	**Population with 1 risk factor**			
2	SBF	4.18	7.92 (4.20 to 11.63)	<.001
3	LSEP	4.04	8.22 (4.23 to 12.20)	<.001
4	TE	2.77	9.48 (2.77 to 16.20)	.006
5	RWG	4.92	9.70 (5.83 to 13.57)	<.001
6	FOwOb	6.87	12.93 (9.24 to 16.62)	<.001
7	MOwOb	5.47	18.26 (11.72 to 24.81)	<.001
	**Population with 2 risk factors**			
8	LSEP+SBF	3.84	9.87 (4.84 to 14.91)	<.001
9	SBF+TE	2.66	11.36 (3.00 to 19.72)	.008
10	SBF+RWG	4.69	11.62 (6.76 to 16.47)	<.001
11	LSEP+TE	2.69	11.77 (3.20 to 20.35)	0.007
12	LSEP+RWG	4.07	12.04 (6.24 to 17.84)	<.001
13	TE+RWG	2.92	13.81 (4.54 to 23.07)	.004
14	FOwOb+SBF	5.37	15.38 (9.76 to 20.99)	<.001
15	FOwOb+LSEP	5	15.91 (9.67 to 22.15)	<.001
16	FOwOb+TE	3.29	18.13 (7.34 to 28.91)	.001
17	FOwOb+RWG	6.3	18.51 (12.75 to 24.26)	<.001
18	MOwOb+SBF	5.06	21.47 (13.15 to 29.79)	<.001
19	MOwOb+LSEP	4.83	22.16 (13.18 to 31.15)	<.001
20	MOwOb+TE	3.28	24.99 (10.07 to 39.91)	.001
21	MOwOb+RWG	5.7	25.47 (16.71 to 34.22)	<.001
22	MOwOb+FOwOb	8.87	32.08 (24.99 to 39.16)	<.001
	**Population with 3 risk factors**			
23	LSEP+SBF+TE	2.69	14.04 (3.82 to 24.25)	.007
24	LSEP+SBF+RWG	4.27	14.35 (7.77 to 20.93)	<.001

25	SBF+TE+RWG	2.95	16.39 (5.51 to 27.26)	.003
26	LSEP+TE+RWG	2.87	16.95 (5.36 to 28.55)	.004
27	FOwOb+SBF+LSEP	4.78	18.80 (11.10 to 26.50)	<.001
28	FOwOb+SBF+TE	3.21	21.32 (8.31 to 34.32)	.001
29	FOwOb+SBF+RWG	6.06	21.74 (14.71 to 28.78)	<.001
30	FOwOb+LSEP+TE	3.23	22.00 (8.66 to 35.35)	.001
31	FOwOb+LSEP+RWG	4.97	22.44 (13.59 to 31.29)	<.001
32	FOwOb+TE+RWG	3.56	25.29 (11.37 to 39.22)	<.001
33	MOwOb+SBF+LSEP	4.99	25.84 (15.69 to 35.98)	<.001
34	MOwOb+SBF+TE	3.35	28.96 (12.00 to 45.91)	.001
35	MOwOb+SBF+RWG	6.07	29.48 (19.96 to 39.01)	<.001
36	MOwOb+LSEP+TE	3.39	29.80 (12.55 to 47.04)	.001
37	MOwOb+LSEP+RWG	5.19	30.33 (18.87 to 41.80)	<.001
38	MOwOb+TE+LSEP	3.72	33.75 (15.99 to 51.51)	<.001
39	MOwOb+FOwOb+SBF	7.9	36.62 (27.54 to 45.71)	<.001
40	MOwOb+FOwOb+LSEP	7.16	37.57 (27.28 to 47.86)	<.001
41	MOwOb+FOwOb+TE	4.58	41.32 (23.62 to 59.02)	<.001
42	MOwOb+FOwOb+RWG	8.81	41.94 (32.61 to 51.26)	<.001
	**Population with 4 risk factors**			
43	LSEP+SBF+TE+RWG	3.02	19.98 (7.00 to 32.97)	.003
44	FOwOb+SBF+LSEP+TE	3.3	25.66 (10.44 to 40.88)	.001
45	FOwOb+SBF+LSEP+RWG	5.4	26.15 (16.65 to 35.64)	<.001
46	FOwOb+SBF+TE+RWG	3.7	29.29 (13.76 to 44.83)	<.001
47	FOwOb+LSEP+TE+RWG	3.56	30.14 (13.53 to 46.75)	<.001
48	MOwOb+SBF+LSEP+TE	3.6	34.18 (15.59 to 52.78)	<.001
49	MOwOb+SBF+LSEP+RWG	6.01	34.76 (23.43 to 46.09)	<.001
50	MOwOb+SBF+TE+RWG	4.03	38.4 (19.74 to 57.06)	<.001
51	MOwOb+LSEP+TE+RWG	3.92	39.36 (19.69 to 59.03)	<.001
52	MOwOb+FOwOb+SBF+LSEP	7.65	42.41 (31.54 to 53.28)	<.001
53	MOwOb+FOwOb+SBF+TE	4.81	46.28 (27.43 to 65.13)	<.001

54	MOwOb+FOwOb+SBF+RWG	9.85	46.91 (37.58 to 56.25)	<.001
55	MOwOb+FOwOb+LSEP+TE	4.86	47.29 (28.20 to 66.38)	<.001
56	MOwOb+FOwOb+LSEP+RWG	7.75	47.92 (35.80 to 60.05)	<.001
57	MOwOb+FOwOb+TE+RWG	5.49	51.85 (33.32 to 70.37)	<.001
	**Population with 5 risk factors**			
58	FOwOb+SBF+LSEP+TE+RWG	3.86	34.55 (17.03 to 52.07)	<.001
59	MOwOb+SBF+LSEP+TE+RWG	4.43	44.27 (24.70 to 63.84)	<.001
60	MOwOb+FOwOb+SBF+LSEP+TE	5.37	52.33 (33.23 to 71.44)	<.001
61	MOwOb+FOwOb+SBF+LSEP+RWG	9.61	52.97 (42.16 to 63.77)	<.001
62	MOwOb+FOwOb+SBF+TE+RWG	6.19	56.85 (38.85 to 74.86)	<.001
63	MOwOb+FOwOb+LSEP+TE+RWG	5.97	57.84 (38.85 to 76.84)	<.001
	**Population with 6 risk factors**			
64	MOwOb+FOwOb+SBF+LSES+TE+RWG	7.06	62.67 (45.27 to 80.07)	<.001

MOwOb: maternal overweight/obesity during the child's infancy; FOwOb: paternal overweight/obesity during the child's infancy; SBF: breastfeeding duration < 6 months; TE: tobacco exposure; RWG: rapid weight gain during infancy; LSES: low family socioeconomic score.

Table S6. Interaction effect of maternal overweight/obesity during the child's infancy and children's breastfeeding duration < 6 months on the risk of overweight/obesity in late childhood.

Combination of risk factors	Unadjusted Model<br>RR (95% CI)	Adjusted Model 1<br>RR (95% CI)	Adjusted Model 2<br>RR (95% CI)
**Primary Outcome for Children**			
**Risk of Overweight or obesity**			
No maternal overweight/obesity or breastfeeding duration < 6 months	1 [Reference]	1 [Reference]	1 [Reference]
Maternal overweight/obesity	1.95 (1.53 to 2.48)	1.84 (1.44 to 2.35)	1.88 (1.46 to 2.41)
Breastfeeding duration < 6 months	0.71 (0.48 to 1.06)	0.73 (0.49 to 1.09)	0.71 (0.47 to 1.06)
Maternal overweight/obesity and breastfeeding duration < 6 months	2.61 (2.06 to 3.31)	2.57 (2.03 to 3.26)	2.53 (1.97 to 3.25)
Additive interaction (95% CI)			
AP	**0.37 (0.17 to 0.56)**	**0.39 (0.20 to 0.58)**	**0.37 (0.17 to 0.57)**
RERI	**0.95 (0.39 to 1.52)**	**1.00 (0.45 to 1.54)**	**0.94 (0.38 to 1.50)**
SI	**2.45 (1.11 to 5.40)**	**2.74 (1.12 to 6.72)**	**2.60 (1.08 to 6.28)**
Multiplicative interaction (95% CI)	**1.88 (1.19 to 2.97)**	**1.91 (1.22 to 3.01)**	**1.93 (1.22 to 3.05)**
**Secondary Outcome for Children**			
**Risk of fat mass index ≥ 75th**			
No maternal overweight/obesity or breastfeeding duration < 6 months	1 [Reference]	1 [Reference]	1 [Reference]
Maternal overweight/obesity	2.57 (1.70 to 3.87)	2.45 (1.62 to 3.69)	2.28 (1.50 to 3.46)
Breastfeeding duration < 6 months	0.92 (0.50 to 1.71)	0.95 (0.51 to 1.77)	0.86 (0.46 to 1.59)
Maternal overweight/obesity and breastfeeding duration < 6 months	4.00 (2.70 to 5.93)	4.00 (2.70 to 5.94)	3.53 (2.34 to 5.32)
Additive interaction (95% CI)			
AP	**0.38 (0.12 to 0.64)**	**0.40 (0.15 to 0.66)**	**0.39 (0.12 to 0.67)**
RERI	**1.51 (0.28 to 2.75)**	**1.61 (0.37 to 2.84)**	**1.39 (0.25 to 2.53)**
SI	**2.02 (1.00 to 4.09)**	**2.15 (1.02 to 4.50)**	2.23 (0.95 to 5.24)
Multiplicative interaction (95% CI)	1.69 (0.84 to 3.43)	1.72 (0.85 to 3.48)	1.82 (0.89 to 3.69)
**Risk of body fat percentage ≥ 75th**			
No maternal overweight/obesity or breastfeeding duration < 6 months	1 [Reference]	1 [Reference]	1 [Reference]
Maternal overweight/obesity	2.07 (1.60 to 2.70)	2.01 (1.54 to 2.61)	1.96 (1.50 to 2.57)
Breastfeeding duration < 6 months	0.94 (0.64 to 1.38)	0.94 (0.64 to 1.39)	0.92 (0.63 to 1.36)
Maternal overweight/obesity and breastfeeding duration < 6 months	2.59 (1.99 to 3.37)	2.53 (1.95 to 3.29)	2.40 (1.82 to 3.17)
Additive interaction (95% CI)			
AP	0.22 (-0.01 to 0.46)	0.23 (-0.01 to 0.47)	0.21 (-0.03 to 0.46)

RERI	0.58 (-0.07 to 1.23)	0.58 (-0.05 to 1.21)	0.52 (-0.11 to 1.14)
SI	1.57 (0.86 to 2.87)	1.61 (0.86 to 3.01)	1.58 (0.81 to 3.09)
Multiplicative interaction (95% CI)	1.33 (0.85 to 2.10)	1.34 (0.85 to 2.10)	1.35 (0.86 to 2.12)
**Risk of waist-to-height ratio ≥ 0.5**			
No maternal overweight/obesity or breastfeeding duration < 6 months	1 [Reference]	1 [Reference]	1 [Reference]
Maternal overweight/obesity	2.86 (1.77 to 4.63)	2.71 (1.67 to 4.40)	2.47 (1.51 to 4.03)
Breastfeeding duration < 6 months	1.20 (0.61 to 2.35)	1.24 (0.63 to 2.42)	1.09 (0.56 to 2.14)
Maternal overweight/obesity and breastfeeding duration < 6 months	4.54 (2.85 to 7.22)	4.53 (2.85 to 7.22)	4.05 (2.51 to 6.56)
Additive interaction (95% CI)			
AP	**0.33 (0.02 to 0.63)**	**0.35 (0.05 to 0.65)**	**0.37 (0.06 to 0.67)**
RERI	1.48 (-0.13 to 3.08)	1.58 (-0.02 to 3.19)	1.50 (0.02 to 2.98)
SI	1.72 (0.88 to 3.35)	1.81 (0.90 to 3.63)	1.96 (0.88 to 4.36)
Multiplicative interaction (95% CI)	1.32 (0.61 to 2.87)	1.35 (0.62 to 2.94)	1.51 (0.69 to 3.29)

Adjusted model 1: adjusted for maternal age at conception, child's sex, age at measurement, and birth weight.

Adjusted model 2: adjusted for maternal age at conception, child's sex, age at measurement, birth weight, delivery by cesarean section (yes or no), assisted reproductive technology for conception (yes or no), time of solid foods introduction (≥ 4 months or < 4 months), maternal smoking during pregnancy (yes or no), and maternal gestational diabetes (yes or no).

AP: attributable proportion; RERI: relative excess risk due to interaction; SI: synergy index. CI: confidence interval.

Table S7. Interaction effect of maternal overweight/obesity and paternal overweight/obesity during the child's infancy on the risk of overweight/obesity in late childhood.

Combination of risk factors	Unadjusted Model RR (95% CI)	Adjusted Model 1 RR (95% CI)	Adjusted Model 2 RR (95% CI)
**Primary Outcome for Children**			
**Risk of Overweight or obesity**			
No maternal overweight/obesity or paternal overweight/obesity	1 [Reference]	1 [Reference]	1 [Reference]
Maternal overweight/obesity	4.00 (2.49 to 6.42)	3.95 (2.47 to 6.33)	4.16 (2.58 to 6.70)
Paternal overweight/obesity	2.41 (1.56 to 3.74)	2.43 (1.57 to 3.76)	2.32 (1.48 to 3.63)
Maternal overweight/obesity and paternal overweight/obesity	4.97 (3.29 to 7.51)	4.76 (3.14 to 7.20)	4.77 (3.12 to 7.29)
Additive interaction (95% CI)			
AP	-0.09 (-0.37 to 0.19)	-0.13 (-0.42 to 0.16)	-0.15 (-0.45 to 0.15)
RERI	-0.44 (-1.87 to 0.98)	-0.62 (-2.04 to 0.80)	-0.70 (-2.20 to 0.79)
SI	0.90 (0.66 to 1.23)	0.86 (0.63 to 1.17)	0.84 (0.61 to 1.16)
Multiplicative interaction (95% CI)	**0.51 (0.30 to 0.87)**	**0.50 (0.29 to 0.84)**	**0.50 (0.29 to 0.84)**
**Secondary Outcome for Children**			
**Risk of fat mass index ≥ 75th**			
No maternal overweight/obesity or paternal overweight/obesity	1 [Reference]	1 [Reference]	1 [Reference]
Maternal overweight/obesity	8.03 (3.03 to 21.30)	7.89 (2.97 to 20.91)	7.80 (2.94 to 20.65)
Paternal overweight/obesity	5.05 (2.01 to 12.68)	5.09 (2.03 to 12.76)	4.87 (1.94 to 12.22)
Maternal overweight/obesity and paternal overweight/obesity	10.95 (4.48 to 26.79)	10.71 (4.38 to 26.21)	9.82 (4.24 to 24.11)
Additive interaction (95% CI)			
AP	-0.10 (-0.53 to 0.33)	-0.12 (-0.55 to 0.32)	-0.19 (-0.66 to 0.28)
RERI	-1.13 (-6.00 to 3.74)	-1.26 (-6.10 to 3.58)	-1.84 (-6.81 to 3.14)
SI	0.90 (0.59 to 1.37)	0.88 (0.58 to 1.35)	0.83 (0.54 to 1.28)
Multiplicative interaction (95% CI)	**0.27 (0.10 to 0.77)**	**0.27 (0.09 to 0.76)**	**0.26 (0.09 to 0.74)**
**Risk of body fat percentage ≥ 75th**			
No maternal overweight/obesity or paternal overweight/obesity	1 [Reference]	1 [Reference]	1 [Reference]
Maternal overweight/obesity	3.19 (1.98 to 5.12)	3.13 (1.95 to 5.02)	3.04 (1.87 to 4.94)
Paternal overweight/obesity	1.94 (1.26 to 2.98)	1.96 (1.27 to 3.01)	1.88 (1.21 to 2.92)
Maternal overweight/obesity and paternal overweight/obesity	3.95 (2.64 to 5.90)	3.87 (2.59 to 5.79)	3.71 (2.45 to 5.60)
Additive interaction (95% CI)			
AP	-0.05 (-0.36 to 0.26)	-0.06 (-0.37 to 0.26)	-0.06 (-0.39 to 0.27)

RERI	-0.18 (-1.42 to 1.05)	-0.21 (-1.43 to 1.00)	-0.22 (-1.44 to 1.01)
SI	0.94 (0.64 to 1.39)	0.93 (0.63 to 1.37)	0.93 (0.61 to 1.40)
Multiplicative interaction (95% CI)	0.64 (0.37 to 1.09)	0.63 (0.37 to 1.08)	0.65 (0.37 to 1.12)
**Risk of waist-to-height ratio ≥ 0.5**			
No maternal overweight/obesity or paternal overweight/obesity	1 [Reference]	1 [Reference]	1 [Reference]
Maternal overweight/obesity	7.08 (1.95 to 25.72)	6.99 (1.92 to 25.39)	6.90 (1.91 to 24.98)
Paternal overweight/obesity	6.26 (1.92 to 20.38)	6.28 (1.93 to 20.42)	6.15 (1.89 to 20.05)
Maternal overweight/obesity and paternal overweight/obesity	16.65 (5.29 to 52.48)	16.35 (5.19 to 51.54)	14.16 (4.47 to 44.82)
Additive interaction (95% CI)			
AP	0.26 (-0.09 to 0.61)	0.25 (-0.10 to 0.60)	0.15 (-0.25 to 0.55)
RERI	4.32 (-2.93 to 11.56)	4.08 (-2.97 to 11.13)	2.11 (-3.90 to 8.11)
SI	1.38 (0.83 to 2.31)	1.36 (0.82 to 2.28)	1.19 (0.71 to 2.00)
Multiplicative interaction (95% CI)	0.38 (0.10 to 1.46)	0.37 (0.10 to 1.44)	0.33 (0.09 to 1.29)

Adjusted model 1: adjusted for maternal age at conception, child's sex, age at measurement, and birth weight.

Adjusted model 2: adjusted for maternal age at conception, child's sex, age at measurement, birth weight, delivery by cesarean section (yes or no), assisted reproductive technology for conception (yes or no), time of solid foods introduction (≥ 4 months or < 4 months), maternal smoking during pregnancy (yes or no), and maternal gestational diabetes (yes or no).

AP: attributable proportion; RERI: relative excess risk due to interaction; SI: synergy index. CI: confidence interval.

Table S8. Interaction effect of paternal overweight/obesity during the child's infancy and low family socioeconomic score on the risk of overweight/obesity in late childhood.

Combination of risk factors	Unadjusted Model RR (95% CI)	Adjusted Model 1 RR (95% CI)	Adjusted Model 2 RR (95% CI)
**Primary Outcome for Children**			
**Risk of Overweight or obesity**			
No paternal overweight/obesity or low family socioeconomic score	1 [Reference]	1 [Reference]	1 [Reference]
Paternal overweight/obesity	2.29 (1.68 to 3.14)	2.20 (1.61 to 3.01)	2.05 (1.50 to 2.82)
Low family socioeconomic score	2.52 (1.66 to 3.81)	2.30 (1.50 to 3.52)	2.07 (1.32 to 3.23)
Paternal overweight/obesity and low family socioeconomic score	2.88 (2.01 to 4.13)	2.64 (1.84 to 3.79)	2.44 (1.68 to 3.54)
Additive interaction (95% CI)			
AP	-0.32 (-0.73 to 0.09)	-0.32 (-0.75 to 0.10)	-0.28 (-0.71 to 0.15)
RERI	-0.93 (-2.08 to 0.22)	-0.86 (-1.95 to 0.24)	-0.68 (-1.71 to 0.35)
SI	0.67 (0.43 to 1.04)	0.66 (0.41 to 1.05)	0.68 (0.40 to 1.14)
Multiplicative interaction (95% CI)	**0.50 (0.31 to 0.81)**	**0.52 (0.32 to 0.85)**	**0.57 (0.35 to 0.96)**
**Secondary Outcome for Children**			
**Risk of fat mass index ≥ 75th**			
No paternal overweight/obesity or low family socioeconomic score	1 [Reference]	1 [Reference]	1 [Reference]
Paternal overweight/obesity	3.22 (1.82 to 5.68)	3.16 (1.79 to 5.58)	2.89 (1.63 to 5.12)
Low Family socioeconomic score	3.60 (1.75 to 7.42)	3.35 (1.61 to 7.00)	2.61 (1.22 to 5.59)
Paternal overweight/obesity and low family socioeconomic score	4.33 (2.29 to 8.19)	4.18 (2.21 to 7.90)	3.34 (1.74 to 6.42)
Additive interaction (95% CI)			
AP	-0.34 (-0.99 to 0.30)	-0.32 (-0.96 to 0.32)	-0.35 (-1.03 to 0.33)
RERI	-1.48 (-4.22 to 1.26)	-1.33 (-3.96 to 1.30)	-1.16 (-3.40 to 1.07)
SI	0.69 (0.38 to 1.26)	0.70 (0.38 to 1.30)	0.67 (0.34 to 1.31)
Multiplicative interaction (95% CI)	**0.37 (0.16 to 0.86)**	**0.39 (0.17 to 0.91)**	0.44 (0.19 to 1.04)
**Risk of body fat percentage ≥ 75th**			
No paternal overweight/obesity or low family socioeconomic score	1 [Reference]	1 [Reference]	1 [Reference]
Paternal overweight/obesity	2.10 (1.52 to 2.91)	2.05 (1.48 to 2.83)	1.83 (1.32 to 2.54)
Low Family socioeconomic score	2.58 (1.69 to 3.94)	2.41 (1.56 to 3.72)	1.81 (1.13 to 2.90)
Paternal overweight/obesity and low family socioeconomic score	2.74 (1.88 to 3.98)	2.60 (1.78 to 3.79)	2.24 (1.52 to 3.29)
Additive interaction (95% CI)			
AP	-0.34 (-0.79 to 0.10)	-0.33 (-0.79 to 0.12)	-0.18 (-0.62 to 0.26)

RERI	-0.94 (-2.14 to 0.25)	-0.86 (-2.02 to 0.29)	-0.40 (-1.37 to 0.57)
SI	0.65 (0.40 to 1.05)	0.65 (0.39 to 1.08)	0.76 (0.41 to 1.40)
Multiplicative interaction (95% CI)	**0.51 (0.31 to 0.83)**	**0.53 (0.32 to 0.87)**	0.68 (0.39 to 1.16)
**Risk of waist-to-height ratio ≥ 0.5**			
No paternal overweight/obesity or low family socioeconomic score	1 [Reference]	1 [Reference]	1 [Reference]
Paternal overweight/obesity	4.00 (2.02 to 7.89)	3.91 (1.98 to 7.72)	3.47 (1.76 to 6.84)
Low Family socioeconomic score	2.59 (0.99 to 6.79)	2.63 (1.01 to 6.89)	1.74 (0.63 to 4.78)
Paternal overweight/obesity and low family socioeconomic score	5.76 (2.75 to 12.08)	5.64 (2.69 to 11.82)	3.94 (1.84 to 8.42)
Additive interaction (95% CI)			
AP	0.03 (-0.48 to 0.54)	0.02 (-0.51 to 0.54)	-0.07 (-0.66 to 0.52)
RERI	0.18 (-2.80 to 3.15)	0.09 (-2.87 to 3.06)	-0.27 (-2.58 to 2.05)
SI	1.04 (0.55 to 1.97)	1.02 (0.53 to 1.95)	0.92 (0.44 to 1.90)
Multiplicative interaction (95% CI)	0.56 (0.19 to 1.59)	0.55 (0.19 to 1.57)	0.65 (0.22 to 1.95)

Adjusted model 1: adjusted for maternal age at conception, child's sex, age at measurement, and birth weight.

Adjusted model 2: adjusted for maternal age at conception, child's sex, age at measurement, birth weight, delivery by cesarean section (yes or no), assisted reproductive technology for conception (yes or no), time of solid foods introduction (≥ 4 months or < 4 months), maternal smoking during pregnancy (yes or no), and maternal gestational diabetes (yes or no).

AP: attributable proportion; RERI: relative excess risk due to interaction; SI: synergy index. CI: confidence interval.

Table S9. Interaction effect of maternal overweight/obesity during the child's infancy and tobacco exposure on the risk of overweight/obesity in late childhood.

	Unadjusted Model	Adjusted Model 1	Adjusted Model 2
**Combination of risk factors**	**RR (95% CI)**	**RR (95% CI)**	**RR (95% CI)**
**Primary Outcome for Children**			
**Risk of Overweight or obesity**			
No maternal overweight/obesity or tobacco exposure	1 [Reference]	1 [Reference]	1 [Reference]
Maternal overweight/obesity	2.54 (2.08 to 3.09)	2.45 (2.01 to 2.99)	2.51 (2.04 to 3.09)
Tobacco exposure	2.28 (1.41 to 3.68)	2.25 (1.40 to 3.60)	2.31 (1.39 to 3.81)
Maternal overweight/obesity and tobacco exposure	3.38 (2.42 to 4.73)	3.03 (2.13 to 4.30)	3.32 (2.31 to 4.78)
Additive interaction (95% CI)			
AP	-0.13 (-0.59 to 0.33)	-0.22 (-0.73 to 0.29)	-0.15 (-0.63 to 0.34)
RERI	-0.43 (-1.90 to 1.03)	-0.67 (-2.09 to 0.74)	-0.49 (-2.00 to 1.02)
SI	0.85 (0.48 to 1.48)	0.75 (0.41 to 1.36)	0.83 (0.46 to 1.49)
Multiplicative interaction (95% CI)	0.58 (0.33 to 1.03)	**0.55 (0.31 to 0.97)**	0.57 (0.32 to 1.03)
**Secondary Outcome for Children**			
**Risk of fat mass index ≥ 75th**			
No maternal overweight/obesity or tobacco exposure	1 [Reference]	1 [Reference]	1 [Reference]
Maternal overweight/obesity	3.34 (2.39 to 4.67)	3.26 (2.33 to 4.55)	3.26 (2.30 to 4.63)
Tobacco exposure	3.74 (1.89 to 7.41)	3.57 (1.79 to 7.14)	3.71 (1.84 to 7.48)
Maternal overweight/obesity and tobacco exposure	6.02 (3.67 to 9.88)	5.54 (3.32 to 9.24)	5.18 (2.97 to 9.04)
Additive interaction (95% CI)			
AP	-0.01 (-0.58 to 0.56)	-0.05 (-0.67 to 0.56)	-0.15 (-0.84 to 0.53)
RERI	-0.06 (-3.50 to 3.37)	-0.29 (-3.62 to 3.04)	-0.80 (-4.10 to 2.51)
SI	0.99 (0.50 to 1.95)	0.94 (0.46 to 1.91)	0.84 (0.41 to 1.73)
Multiplicative interaction (95% CI)	0.48 (0.21 to 1.09)	0.48 (0.21 to 1.09)	**0.43 (0.19 to 0.98)**
**Risk of body fat percentage ≥ 75th**			
No maternal overweight/obesity or tobacco exposure	1 [Reference]	1 [Reference]	1 [Reference]
Maternal overweight/obesity	2.51 (2.03 to 3.10)	2.46 (1.99 to 3.04)	2.44 (1.95 to 3.04)
Tobacco exposure	2.90 (1.87 to 4.50)	2.78 (1.79 to 4.33)	2.83 (1.78 to 4.50)
Maternal overweight/obesity and tobacco exposure	3.57 (2.52 to 5.07)	3.31 (2.30 to 4.76)	3.31 (2.24 to 4.89)
Additive interaction (95% CI)			
AP	-0.23 (-0.74 to 0.27)	-0.28 (-0.83 to 0.26)	-0.29 (-0.85 to 0.27)

RERI	-0.83 (-2.49 to 0.82)	-0.94 (-2.54 to 0.67)	-0.96 (-2.61 to 0.68)
SI	0.75 (0.44 to 1.31)	0.71 (0.40 to 1.27)	0.71 (0.39 to 1.28)
Multiplicative interaction (95% CI)	**0.49 (0.28 to 0.85)**	**0.48 (0.28 to 0.84)**	**0.48 (0.27 to 0.84)**
**Risk of waist-to-height ratio ≥ 0.5**			
No maternal overweight/obesity or tobacco exposure	1 [Reference]	1 [Reference]	1 [Reference]
Maternal overweight/obesity	3.57 (2.44 to 5.22)	3.48 (2.38 to 5.08)	3.36 (2.27 to 5.00)
Tobacco exposure	3.02 (1.25 to 7.31)	2.98 (1.24 to 7.21)	2.96 (1.21 to 7.20)
Maternal overweight/obesity and tobacco exposure	6.32 (3.57 to 11.18)	5.62 (3.10 to 10.19)	4.74 (2.48 to 9.04)
Additive interaction (95% CI)			
AP	0.11 (-0.47 to 0.70)	0.13 (-0.64 to 0.69)	-0.12 (-0.91 to 0.66)
RERI	0.72 (-3.21 to 4.66)	0.16 (-3.63 to 3.95)	-0.58 (-4.09 to 2.93)
SI	1.16 (0.52 to 2.56)	1.04 (0.45 to 2.39)	0.87 (0.36 to 2.07)
Multiplicative interaction (95% CI)	0.59 (0.21 to 1.62)	0.54 (0.19 to 1.52)	0.48 (0.17 to 1.33)

Adjusted model 1: adjusted for maternal age at conception, child's sex, age at measurement, and birth weight.

Adjusted model 2: adjusted for maternal age at conception, child's sex, age at measurement, birth weight, delivery by cesarean section (yes or no), assisted reproductive technology for conception (yes or no), time of solid foods introduction (≥ 4 months or < 4 months), maternal smoking during pregnancy (yes or no), and maternal gestational diabetes (yes or no).

AP: attributable proportion; RERI: relative excess risk due to interaction; SI: synergy index. CI: confidence interval.

Table S10. Comparison of characteristics between the imputed and analytic data.

Characteristic	Imputed data (N=1874)	Available data (N=942)	*P-value*
**Mothers**			
Age (years)	31.37 ± 4.88	31.74 ± 4.57	.05
BMI during the child's infancy (kg/m^2)	25.32 ± 5.42	24.98 ± 4.96	.11
College graduate or higher	1460 (77.93)	749 (79.60)	.33
Gestational diabetes (n, %)			.44
Yes	110 (5.85)	48 (5.16)	
No	1764 (94.14)	883 (94.84)	
**Fathers**			
BMI during the child's infancy (kg/m^2)	26.87 ± 4.75	26.73 ± 3.74	.42
**Child**			
Female	919 (49.04)	473 (50.21)	.56
Age (years)	11.50 ± 0.51	11.58 ± 0.50	.02
Birthweight (grams)	3443.58 ± 590.74	3437.64 ± 555.90	.79
Delivered by cesarean section (n, %)			.68
Yes	560 (29.89)	274 (29.12)	
No	1314 (70.11)	667 (70.88)	
Infants with assisted reproductive technology (n, %)			.67
Yes	135 (7.21)	72 (7.65)	
No	1739 (92.79)	869 (92.35)	
Breastfeeding duration (n, %)			.14
≥ 6 months	1196 (63.82)	628 (66.67)	
< 6 months	678 (36.18)	314 (33.33)	
Time of solid foods introduction (n, %)			.17
≥ 4 months	1750 (92.21)	869 (93.64)	
< 4 months	124 (7.79)	59 (6.36)	
Tobacco exposure (n, %)			.05

Yes	121 (9.35)	44 (4.78)	
No	1753 (93.54)	897 (95.22)	
Increment of weight z-score during infancy	0.41 ± 1.27	0.32 ± 1.19	.07
BMI at 11-12 years (kg/m²)	19.22 ± 3.43	19.10 ± 3.35	.40
FMI at 11-12 years (kg/m²)	4.42 ± 2.61	4.33 ± 2.57	.39
BF% at 11-12 years (%)	21.71 ± 8.41	21.43 ± 8.41	.39
WHtR at 11-12 years	0.43 ± 0.05	0.43 ± 0.05	.10
Family socioeconomic score	0.31 ± 0.95	0.42 ± 0.91	.01

BMI: body mass index; FMI: fat mass index; BF%: body fat percentage; WHtR: waist-to-height ratio.

Table S11. Associations of the postnatal risk factor scores with overweight/obesity in children aged 11-12 years: finding from imputed data (n=1874).

	Unadjusted model		Adjusted model 1		Adjusted model 2	
**Number of risk factors**	**RR (95% CI)**	***P-value***	**RR (95% CI)**	***P-value***	**RR (95% CI)**	***P-value***
**Primary Outcome for Children**						
Overweight or obesity						
2 risk scores	2.00 (1.44 to 2.76)	<.001	2.00 (1.45 to 2.77)	<.001	2.03 (1.47 to 2.79)	<.001
3 risk scores	2.49 (1.82 to 3.42)	<.001	2.52 (1.84 to 3.46)	<.001	2.50 (1.79 to 3.49)	<.001
≥ 4 risk scores	3.58 (2.63 to 4.87)	<.001	3.67 (2.70 to 4.99)	<.001	3.66 (2.65 to 5.07)	<.001
P-for-trend	<.001		<.001		<.001	
**Secondary Outcome for Children**						
Fat mass index (≥ 75th) [b]						
2 risk scores	2.16 (1.24 to 3.75)	.01	2.17 (1.25 to 3.77)	.01	2.21 (1.26 to 3.87)	.01
3 risk scores	3.30 (2.00 to 5.43)	<.001	3.35 (2.03 to 5.51)	<.001	3.16 (1.85 to 5.38)	<.001
≥ 4 risk scores	5.45 (3.38 to 8.81)	<.001	5.60 (3.46 to 9.05)	<.001	5.31 (3.17 to 8.89)	<.001
P-for-trend	<.001		<.001		<.001	
Body fat percentage (≥ 75th) [b]						
2 risk scores	1.92 (1.36 to 2.71)	<.001	1.92 (1.36 to 2.71)	<.001	1.95 (1.37 to 3.18)	<.001
3 risk scores	2.40 (1.73 to 3.34)	<.001	2.40 (1.72 to 3.34)	<.001	2.32 (1.64 to 3.28)	<.001
≥ 4 risk scores	3.59 (2.60 to 4.91)	<.001	3.60 (2.63 to 4.93)	<.001	3.55 (2.53 to 4.98)	<.001
P-for-trend	<.001		<.001		<.001	
Waist-to-height ratio (≥ 0.5)						
2 risk scores	2.32 (1.22 to 4.39)	.01	2.36 (1.25 to 4.48)	.01	2.31 (1.16 to 4.61)	.02
3 risk scores	4.12 (2.34 to 7.23)	<.001	4.17 (2.37 to 7.33)	<.001	3.81 (2.04 to 7.12)	<.001
≥ 4 risk scores	6.50 (3.71 to 11.41)	<.001	6.75 (3.84 to 11.87)	<.001	6.13 (3.34 to 11.25)	<.001
P-for-trend	<.001		<.001		<.001	

All effect estimates are referenced to children with 0-1 risk factor (n = 354). Six risk factors were: maternal and paternal overweight/obesity during the child's infancy, low family socioeconomic score, breastfeeding duration< 6 months, rapid weight gain during infancy, and tobacco exposure.

[a] Age- and sex-specific BMI z scores were calculated using the CDC sex-specific BMI-for-age growth charts from 2000, being a healthy weight was defined as having a BMI z score < 85th percentile, and being overweight/obese was defined as having a BMI z scores ≥ 85th percentile for age and sex.

[b] Participants with an FMI and/or BF% greater than or equal to the age- and sex-specific 75th percentile was classified as overweight/obese, according to published reference Curves for American NHANES IV for BF% and FMI.

Adjusted model 1: adjusted for maternal age at conception, child's sex, age at measurement, and birth weight.

Adjusted model 2: model 1+ adjusted for the child's gestational age, delivery by cesarean section (yes or no), assisted reproductive technology for conception (yes or no), time of solid foods introduction (≥ 4 months or < 4 months), maternal smoking during pregnancy (yes or no), and maternal gestational diabetes (yes or no).

RR: relative risk. CI: confidence interval.

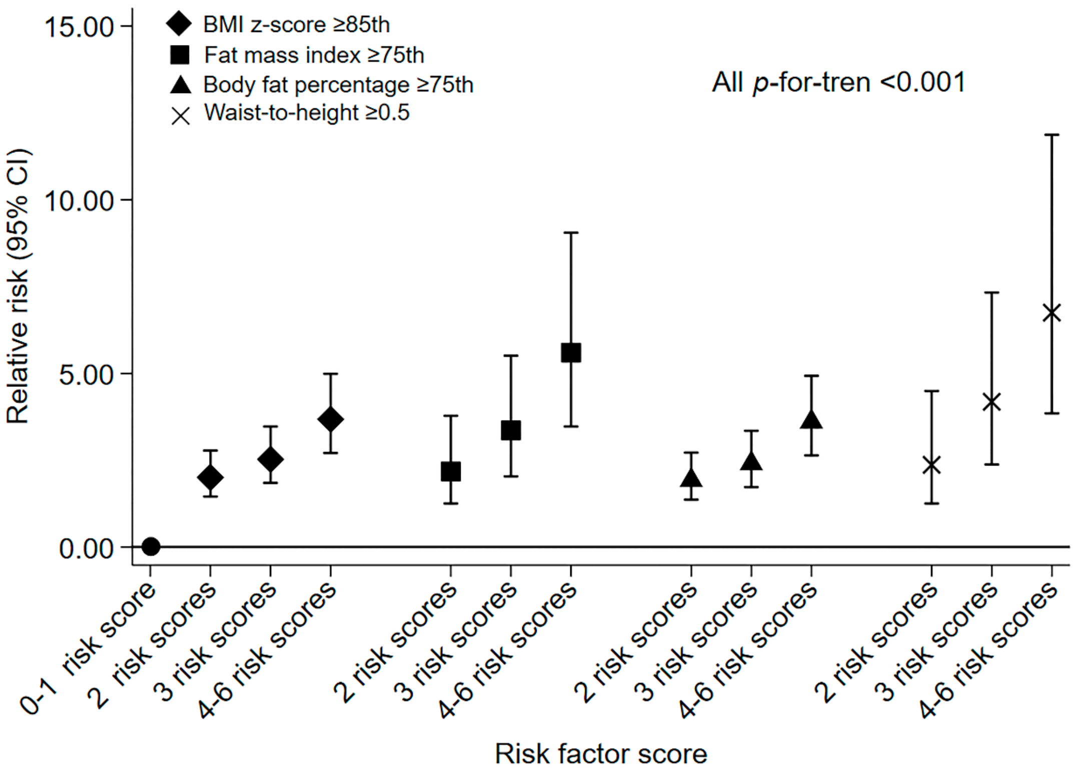

Figure S1. Associations of the postnatal risk factor scores with overweight/obesity risks in children aged 11-12 years: findings from imputed data (n=1874). All modes were adjusted for the child's sex, age, birth weight, and maternal age at conception. "●" indicated the baseline reference for the four outcomes. BMI: body mass index.
